# The Role of the Microbiota–Gut–Brain Axis and Antibiotics in ALS and Neurodegenerative Diseases

**DOI:** 10.3390/microorganisms8050784

**Published:** 2020-05-23

**Authors:** Mark Obrenovich, Hayden Jaworski, Tara Tadimalla, Adil Mistry, Lorraine Sykes, George Perry, Robert A. Bonomo

**Affiliations:** 1 Research Service, Louis Stokes Cleveland, Department of Veteran’s Affairs Medical Center, Cleveland, OH 44106, USA; hayden.jaworski@yahoo.com (H.J.); txt214@case.edu (T.T.); Robert.Bonomo@va.gov (R.A.B.); 2 Departments of Chemistry, Biochemistry, Pathology and Molecular Biology, Case Western Reserve University, Cleveland, OH 44106, USA; 3 The Gilgamesh Foundation for Medical Science and Research, Cleveland, OH 44116, USA; 4 Department of Medicinal and Biological Chemistry, College of Pharmacy and Pharmaceutical Sciences, University of Toledo, Toledo, OH 43606, USA; 5 Cleveland State University Departments of Chemistry and Engineering, Cleveland, OH 44115, USA; adilamistry@gmail.com; 6 Department of Laboratory Medicine, Metro Health Medical Center, Cleveland, OH 44109, USA; lorrainneskykes11@gmail.com; 7 Department of Biology University of Texas San Antonio, San Antonio, TX 78249, USA; perry2500@gmail.com

**Keywords:** amyotrophic lateral sclerosis, ALS, chemobiotics, probiotics, symbiotics, neurodegeneration, Alzheimer disease, AD, holobiota, holobiome, microbiota, microbiome, microbiota–gut–brain axis, ceftriaxone

## Abstract

The human gut hosts a wide and diverse ecosystem of microorganisms termed the microbiota, which line the walls of the digestive tract and colon where they co-metabolize digestible and indigestible food to contribute a plethora of biochemical compounds with diverse biological functions. The influence gut microbes have on neurological processes is largely yet unexplored. However, recent data regarding the so-called leaky gut, leaky brain syndrome suggests a potential link between the gut microbiota, inflammation and host co-metabolism that may affect neuropathology both locally and distally from sites where microorganisms are found. The focus of this manuscript is to draw connection between the microbiota–gut–brain (MGB) axis, antibiotics and the use of “BUGS AS DRUGS” for neurodegenerative diseases, their treatment, diagnoses and management and to compare the effect of current and past pharmaceuticals and antibiotics for alternative mechanisms of action for brain and neuronal disorders, such as Alzheimer disease (AD), Amyotrophic Lateral Sclerosis (ALS), mood disorders, schizophrenia, autism spectrum disorders and others. It is a paradigm shift to suggest these diseases can be largely affected by unknown aspects of the microbiota. Therefore, a future exists for applying microbial, chemobiotic and chemotherapeutic approaches to enhance translational and personalized medical outcomes. Microbial modifying applications, such as CRISPR technology and recombinant DNA technology, among others, echo a theme in shifting paradigms, which involve the gut microbiota (GM) and mycobiota and will lead to potential gut-driven treatments for refractory neurologic diseases.

## 1. Introduction

The human “microbiota” or the more novel name “holobiota”, is considered to comprise the entire and diverse collection of microbes, mycobiota, parasites, pathogens, commensals, viruses, viroids, protozoan parasites, their individual and collective genomes, coding and noncoding nucleic acids, and the co-metabolism between any of these exogenous microorganism that live within the human gut or elsewhere. The microbiota, contain both disease causing and disease preventing organisms and while in the public domain, i.e., in all of our collective bowels, microbes are considered drugs by the government regulators and not propitious probiotics, which they actually could become. Whether they are “bugs as drugs” or harmless symbionts of our digestive system, they hold the key to understanding and manipulating our health and disease propensity by linking the most elusive diseases known to standard medicine and modern practice to gut bacterial profiles. This is a transformative concept for the protractive disease, particularly the neurodegenerative ones.

In regard to the holobiome, important environmental factor is growing reports it exerts an influence on symptoms and the fraction making up the smallest percentage, namely 1%, have a disproportionately large effect on disease [1]. Emerging studies now show some neurodegenerative diseases, particularly, Alzheimer disease, Multiple Sclerosis, Parkinson disease and amyotrophic lateral sclerosis, have specific microbiome profiles or a disparity among select microbes that seemingly match the corresponding diagnosed pathology in human subjects [2]. Many proposed therapeutic treatments revolve around the idea of restoring normal gut flora, either through fecal transplant, antibiotics or through psychobiotics administration. The role that antibiotics play in these correlations has been of recent interest in neurological research. It has been hypothesized that impaired gut integrity and reduced gut homeostasis has an active role in ALS and other neurologic diseases, which are most difficult for standard medicine. We speculate that this may be in part due to the neglect the human physiology has for our second genome and the microbial contribution to our biochemistry.

Neurodegenerative diseases are diverse disorders that are characterized by progressive loss of neurons, neuronal structure and cellular function, which occurs in distinct sections of the central nervous system and brain. Alzheimer disease, which is the most common neurodegenerative disease, is particularly devastating. As with most neurodegenerative diseases, it is progressive, has human essence-robbing characteristics, leads to declines in cognitive capacity, involves multiple facets of dementia and leads to eventual death. Although the mechanisms of AD pathogenesis are still being explored and understood, the newer emerging trends point to the viruses and GM as a chief players in disease pathogenesis. Contrary to the overuse of broad-spectrum antibiotics that are used to kill off a range of specific microorganisms, probiotics, and those microorganisms yet to be developed or approved for human use, can be considered medicine -aptly deemed “bugs as drugs”. Probiotics, which contain specific strains of microbes are designed to have therapeutic efficacy and have a safety factor for their human use even if they reside in the gut of most humans.

The major function of commensals in a healthy gastrointestinal tract is to resist colonization and proliferation of any potentially pathogenic microbes contained within, which we call colonization resistance [3]. Safe use of probiotics is heavily supported by a variety of studies showing how our endogenous microbes play a major role in the health of an individual, especially in relation to neurological diseases. New studies show specific strains of microorganisms can exhibit positive results, when acting as a probiotic or symbiotic. In recent animal studies, *Lactobacillus* or *Bifidobacterium*, have been employed to investigate the capacity of probiotics and have shown some efficacy in treating neurologic and other diseases [4]. These strains of bacteria have been shown to modulate the production of brain metabolites γ-aminobutyric acid (GABA) and glutamate, which are associated with brain signaling in memory forming regions of the brain [5]. Microbes and the GABA inhibitory neurotransmitter play roles in neuropsychiatric illness and can lead to anxiety and depression [5]. The use of probiotics for the treatment and prevention of Parkinson disease (PD) and AD is still largely unexplored. However, it is highly plausible that probiotics would have beneficial effects on AD and PD through protective small molecule production and through the modulation of inflammatory responses [6], which ultimately lead to oxidative stressors that precipitate a cascade of downstream effects and induce pathogenesis in AD.

Assaults on vulnerable neurons occur frequently and any subsequent inflammation is a part of the wound-healing response. However, cytokine stress contributes to damage. These pro-inflammatory immune responses are associated with natural killer cell circulation and modifications in immune response mechanisms, which lead to specific cytokine production and probiotics improves immune cell balance for macrophages, granulocytes and T cells [4]. Pathogenic bacteria produce pro-inflammatory components and fluctuations in the GM that produce lipopolysaccharide (LPS), for example, which contributes to systemic inflammation and increased LPS load. It is known that LPS crosses immune barriers and results in inflammation [7], which also feeds forward increased permeability of our immune privileged barriers. While extensive dysbiosis is a hallmark of neurodegenerative diseases, such as AD and PD, overgrown populations, nonetheless, may contribute significantly to pathogenicity across the MGB axis in ALS, which also causes intestinal inflammation and lead to increased gut permeability [8]. Gram-negative bacteria in particular have cell membranes, in part comprised of endotoxic LPS, which interact with intestinal cells to cause the release of inflammatory cytokines. This cascade also creates oxidative conditions in the brain and propagates neurodegeneration [9]. Discussed in detail later, the brain’s immune cells, microglia, are also activated by LPS, which are involved then in astrocyte transformation and contribute to cytokine stress by releasing inflammatory molecules [10,11].

These protective systems can be circumvented and evaded by many pathogens, which exhibit immune privilege like *Ebola* virus, which was found to travel along neuronal structures within the retina and escape immune surveillance. Likewise, bacteria, spirochetes and other pathogens can exploit this same vulnerability in the brain, spinal cord and eye because it is believed that a full immune response to infection would be deleterious to sensitive brain and ocular tissues. Another example is the spirochete, *Borrelia burgdorferi*, that causes Lyme neuroborreliosis. It attacks the nervous system as well as eyes and can mimic Parkinson-like symptoms, i.e., confusion and cognitive impairment. Yet another offender, a complex structure adhering to surfaces, known as Biofilm, which consists of mucopolysaccharides, calcium (Ca^2+^) and extracellular DNA, has been suspected of helping bacteria to evade the immune system [12]. It is then of great interest to be able to specifically modulate bacterial populations, minimize overgrown and decrease endotoxic and excitotoxic bacterial strains or protect and propagate probiotics. Probiotics provide colonization resistance against those pathogenic bacteria that would contribute to neurodegeneration and metabolite synthesis that would benefit neuroprotection.

Many examples exist for bacterial component translocation and bacteria breech of our immune barriers. Although not specifically addressed in this review, an oral bacteria, *Porphyromonas gingivalis*, found in brains of neurodegenerative disease patients can access brain compartments and are propagated via several pathways including direct infection, infection of monocytes and endothelial cells, after damaging blood brain barriers (BBB) or through infection that spreads via olfactory or trigeminal cranial nerves [13,14,15]. Regardless of the pathogenesis of neurodegeneration, examples such as these lead us to conclude that although neurologic diseases are multifactorial they have a relationship to our microbiota [14] and the integrity of the gut-blood, BBB and perhaps other immune privileged barriers [13] as well. In that regard, recent evidence suggests that the microbiota may be influencing the pathogenesis of AD, PD and ALS through molecular mimicry pathways. For example, the peptidoglycan recognition protein gene *PGLYRP*, when disrupted or mutated, is linked to PD. This gene in PD has been correlated with low *Prevotellaceae* levels and higher levels of Gram-negative bacteria *Enterobacteriales*, which could indicate dysbiosis over typical *enterococci* sp. levels [16]. The authors of this study successfully used these bacterial species as animal probiotic models and to recapitulate gut dysfunction and stimulate disease progression. As an example, several intracellular protozoan parasites are implicated in AD, specifically *Toxoplasma gondii*, which crosses the BBB and causes neurological dysfunction and encephalitis by promoting chronic inflammation of the brain, meninges and central nervous system (CNS) [14].

In order to make legitimate claims regarding the usefulness of probiotic therapeutic applications for neurodegenerative diseases, larger scale human studies are necessary as much information is still missing or underdeveloped in this area. Nevertheless, to date, there is no cure or effective disease-modifying agents available for most, if not all, neurodegenerative disorders and therapy is largely palliative or focused on symptomatic management [17]. What we do know about the pathogenesis of neurodegeneration is that neurodegenerative diseases, including but not limited to AD, share features and are strongly correlated with oxidative stress, neuroinflammation and accumulation of amyloid-beta (Aβ) plaques or neurofibrillary tangles [18]. While these hallmark end-stage lesions are associated with neurodegenerative diseases, targeting them, i.e., efforts to remove them from human brains, has not resulted in cures or even helped delay disease progression. In those early days, the author and colleagues had cautioned against effort to create antibodies against endogenous proteins, especially in the brain as brain inflammation is extremely deleterious to the very sensitive and vulnerable neurons [19]. At the same time, most of those founding companies created to remove Aβ have failed. When we consider the billions of dollars expended following the amyloid or tau hypotheses, one may argue they were lost targeting lesions and not prodromal mechanisms. While we cannot fault science, we can argue that those who fail to see the alternative or entertain any hypothesis differing from their own, do not really engage in science but are largely chasing money. We, therefore, have turned to novel findings and other promising approaches. These appear on the surface to be distally related, but in fact, they share micro-organismal components to their pathogenesis, including the mitochondrial, the vascular and others [20,21]. One more promising approach is to explore neurodegenerative diseases and their underpinnings that intersect with the microbiota–gut–brain axis. This will most likely aid in our understanding of disease mechanisms and suggest symbiotic combinatorial approaches that could become new standards of care or treatment for these devastating diseases [22,23].

Significantly, many of the worst neurodegenerative diseases, like Alzheimer disease, Parkinson disease and amyotrophic lateral sclerosis, hold a closer association with the GM than previously thought [22]. We have an imperative to explore these relationships with our microbiota and the propagation of healthy protective commensal forms through nutrition or other interventions including selective, appropriate, stewarded and responsible use of antibiotics or inhibitory chemicals if necessary. Moreover, our immune tissue barriers, which, by design, maintain proper homeostasis in vulnerable organs and tissues, prevent foreign substances from entering the systemic circulatory system. These barriers may be more compromised than we once understood or was believed by conventional dogma [13]. We should also consider the small molecules produced by the gut microbiota as a recent mouse study investigating the role of butyrate levels on gut barrier integrity has found. Increasing butyrate is associated with increased barrier homeostasis and longer life expectancy. The ALS drug Triheptanoin was found to protect motor neurons and delay the onset of motor symptoms in a mouse model of ALS also increased levels of plasma β-hydroxybutyrate 1.7 fold [24]. Another example, the introduction of butyrate is believed to stimulate the growth of bacterial strains, such as *Butyrivibrio*, *Bacteroides* and *Odoribacter* [25] and studies using 693A mice, found 2% butyrate in water improved life span. The microbial fermentation by-products are short chain fatty acids (SCFAs), particularly acetate, propionate, and butyrate [26]. As part of a symbiosis, prebiotics act synergistically to modify colonic and intestinal microbiota, to benefit human health [27]. Moreover, an author of the present review found protective aspects of butyrate from potato starch prebiotics with *Faecalibacterium prausnitzii on Clostridium difficile* infection and attenuation of subsequent damage [28,29].

## 2. ALS and Intractable Neurologic Diseases

The intractable diseases, particularly the neurodegenerative ones, pose particular obstacles to success and we have not done well with them as the overall mortality rate for neurological diseases between 1990 and 2015 has actually increased by 37% [30]. One particularly intractable neurologic disease is amyotrophic lateral sclerosis. ALS is more commonly referred to as Lou Gehrig’s disease, who had the disease and why its apostrophized, which is an extremely fatal neurodegenerative disease, a motor neuron disease that causes cell death leading to loss of voluntary and involuntary muscle action.

ALS is rapid, it progressively affects upper and lower motor neurons, results in neuronal loss, degeneration and damage, which leads to breakdown of nerve cells in the brain, spinal cord and in motor neurons. This progressive disorder belongs to a sub-group of neurological pathologies called motor neuron diseases that eventually renders patients progressively debilitated or worse. The associated cell death leads to loss of voluntary and involuntary muscle action. The shared symptoms include muscle weakness, cramping, problems with coordination, stiff muscles, muscle spasms and muscle twitching. The gradual deterioration of neurons in the central nervous system leads to the loss of muscle function and paralysis of both voluntary and involuntary muscles [31]. Gradually, patients have difficulty speaking, swallowing and eventually breathing [32]. ALS belongs to a wider group of disorders known as motor neuron diseases, which are caused by gradual deterioration, degeneration and death of motor neurons only as opposed to all nerve cells in the brain and spinal cord as mentioned by the authors [31,32]. Motor neurons are nerve cells that extend from the brain to the spinal cord and to muscles throughout the body. These motor neurons initiate and provide vital communication links between the brain and the voluntary muscles.

Most cases are diagnosed based on symptoms, physical signs, progression, electromyography and tests to exclude the overlapping conditions [33]. Prominent symptoms of ALS revolve around muscle cramping and weakness. Current medical protocol utilizes the ALS Functional Rating Scale-Revised (ALSFRS-R) to assess twelve tasks, such as speech, salivation, swallowing and writing to elucidate the severity of the ALS symptoms. ALS tends to affect individuals aged forty to seventy and has two known forms, one is Sporadic ALS (SALS), the most common form and the other is Familial ALS (FALS), which has a genetic component. FALS only represents 5–10% of ALS cases. The median survival is around two to five years and variation does occur but 5–10% of patients do survive beyond 10 years [34,35,36]. Approximately twenty percent of individuals with ALS will also develop Frontal Temporal Dementia. Since most patients are of the sporadic form, ALS patients do not have a clear genetic predisposition to develop the disease and thus genetics likely play a limited role [34]. Therefore, exploring the microbiota–gut–brain neuroendocrine axis is important for understanding the pathogenesis of the disease. ALS patients, who carry mutations or gene variants can exhibit subtly distinct clinical features. At the time of publishing, over twenty-five genes, putative or causative, have been identified in hereditary or familial ALS [37], sporadic ALS or both [38].

To date we can summarize genetic findings generally where Familial ALS cases show high heritability and the identification of several genetic associations have been made, in particular they are most frequently mutations or polymorphisms in Super Oxide Dismutase-1 (SOD-1), RNA-binding protein Sarcoma/Translocated in Liposarcoma or FUS/TLS, Trans-activation response (TAR) DNA Binding Protein and chromosome nine open reading frame (C9ORF72) [39,40]. However, these findings translate to only a small fraction of Sporadic ALS, where the etiology of most cases remain unexplained. Here, we review past and current methods used for the identification of FALS and SALS associated genes and propose a risk-based classification for these [41]. These authors outline the major ALS-related genes and genotype–phenotype correlations of ALS. It is important to dissect the genetic factors associated with amyotrophic lateral sclerosis.

Approximately 10% of ALS cases are related to genetics. The first suspected gene associated with ALS was reported to be *SOD-1*, and since then approximately twenty-five additional genes have been explored in connection to ALS pathogenesis. These genes fall into three groups: (1) those genes altering proteostasis, (2) those genes affecting RNA stability and (3) those that disrupt normal cytoskeleton function. Among proteostasis genes are *SOD-1, SQSTM1, VAPB, VCP, UNC13A, DAO, OPTN, UBQLN2, CHCHD10, MATR3, TBK1, SCFD1, NEK1 and C21ORF2.* The genes that affect RNA properties include, *ANG, TARDBP, FUS, ATXN2, C9ORF72, TAF15, and HNRNPA1.* Finally, the genes that affect cytoskeleton properties include, *DCTN1, PFN1, TUBA4A, and MOBP* [28]. Among those reported, *SOD-1* and *C9ORF72* have demonstrated to be most consistently present in genetic ALS cases. *SOD-1* mutations are evident in approximately 22% of ALS cases, with 20% being genetically linked. Likewise, *C9ORF72* appears in roughly 35% of cases, with 25% showing genetic links association with intronic expansions. Trinucleotide expansions of the CAG sequence in the *ATXN2* gene sequence has shown significant susceptibility to ALS pathogenesis. Mutations in *ATXN2* tend to show early motor atrophy. Some additional genes of interest include *TARDBP*, *FUS*, *HNRNPA1*, *SQSTM1*, *VCP*, *OPTN*, *PFN1,* however associations with ALS are not as well defined. The majority of other genetic links are evident in between 1% and 5% of cases [42].

## 3. Antibiotics, Symbiotics and Chemotherapeutics for ALS and Neurodegeneration

Current medications prescribed for ALS include Riluzole and Edaravone [43,44]. While there is no cure for ALS and neurodegenerative diseases, there are some treatments and many studies suggest a strong connection exists between microbiota dysbiosis and neurodegeneration [45]. It is at this node where we turn our attention and focus on the microbiota, not as hype but as transformative, with capabilities to infer that antibiotics and probiotics may have efficacy in treating neurodegenerative diseases, especially where the microbiota–gut–brain axis is involved [23]. However, emerging antibiotic resistance and the overuse of antibiotics in clinical practice and with raising livestock, pose particular problems to our health and the health of our microbiota. Antibiotic stewardship aims to curb some antibiotic misuse, but what is not known is how co-metabolism with the host and the optimal good microbial milieu, or “eubiota”, could be enhanced by antibiotics or natural compounds from food.

While not an ode to antibiotics, we highlight their novel use in the recent past for treating neurodegenerative conditions. In particular, the beta-lactam, cephalosporin antibiotics, such as Ceftriaxone (CFT) and others, have shown efficacy in treating and alleviating neurologic-based disease symptoms and the experimentally-induced neurological disorders Parkinson disease, Alzheimer disease, ALS, traumatic brain injury (TBI), epilepsy, brain ischemia and neuropathy [2,46,47]. CFT has been reported to have neuroprotective and anti-inflammatory effects.

Few drugs have shown effectivity in treating ALS or its symptoms and progression (for a current list see [42]). Chemobiotic therapy or antibiotics and probiotics are increasingly gaining interest in the scientific community. This interest is due to multiple mechanisms of action for antibiotics aimed at relieving symptoms and modifying the natural history and course of various neurodegenerative diseases. Over the past decade, evidence to support use of antibiotics or probiotics and symbiotics to treat neurodegenerative diseases has grown to include clinical trials and limited use in complementary alternative practice or clinical use of probiotics and fecal transplantation material (FTM) to deal with neurodegenerative and other related diseases.

Fecal transplantation has been documented to help restore gut integrity for many conditions including natural immune response, gut-immune normalization and reconditioning. For example, the Fecal Microbiota Transplantation Effect on Amyotrophic Lateral Sclerosis Patients (FETR-ALS) study protocol is a randomized clinical trial using fecal FTM in ALS [48]. This group has shown recently an association between ALS and the immune system, particularly the glia, microglia and regulatory T (T-reg) lymphocytes, which are of particular interest in ALS and other neurodegenerative disorders [11]. The microbiota-gut directly influence T-regs by affecting cell numbers and downstream immune responses involving these immune cells. Previous studies by this group and others demonstrate a correlation between gut dysbiosis and early pre-onset ALS. However, the extent to which these immune cells are active in the progression of ALS is still not well-characterized. Nevertheless, this correlation indicates a potential link between gut microbiota and ALS pathogenesis. This study is first to propose the use of fecal microbial transplantation as a means of therapeutic relief and for the restoration of the gut microbiota in ALS. It is plausible that through the use of FTM, an increase in T-reg cell counts could be observed, as well as improved response in ALS patients. Likewise, some suggest that broad spectrum antibiotics may cause or contribute to neurodegenerative diseases by disrupting normal microbial homeostasis.

Recent studies suggest that, through gut dysbiosis, antibiotics may be influencing multiple sclerosis and ALS pathogenesis. In that regard, a Swedish study on antibiotic use explored the subsequent risk of developing ALS after broad spectrum antibiotics were prescribed [49]. Bacterial analysis and drug register rankings were applied to antibiotics administered years prior to diagnosis of ALS and resulting ALS conditions. This study suggests a connection between microbiota dysbiosis and neurodegenerative diseases and with ALS and antibiotic use. Antibiotics have been known to affect up to 30% of regular microbiota abundance. In the Swedish case studies, ALS patients (*n* = 2484) were examined in order to investigate the correlation between antibiotic use in years prior to diagnosis, and resulting ALS conditions. Using the Swedish Prescribed Drug Register, and bacterial analysis, rankings were able to be applied to the antibiotics administered to each individual in the case study in relation to how they affect the microbiota. Results showed that ALS patients were more likely to receive antibiotics prior to their index dates as compared to control Swedish populations [49]. When excluding antibiotics prescribed within a one-year margin of ALS diagnosis, it was observed that any antibiotic use was associated with increased risk of ALS. Among all associations, it was noted that beta-lactamase sensitive penicillin held a very significant association as compared to other drugs [49].

## 4. History of Antibiotic Use for ALS

What brought us to use antibiotics in the first place may hold the key to future treatments. Clinically, CFT, tetracycline and other beta-lactam drugs have been used to treat ALS. While we are not postulating an old story, the first identified use for CFT for the treatment of ALS was in 1988, when a study was undertaken to explore a relationship between ALS and Lyme disease. Lyme, also known as *Borrelia burgdorferi* infection, was correlated with ALS when anti-*Borrelia* antibodies were found in ALS patients [49]. Thus, a course of Ceftriaxone was tried because the drug had recently been found to be active against *Borrelia* [50]. In that study, serum samples from ALS patients were positive for anti-*Borrelia* antibodies by anti-*Borrelia* immunofluorescent antibody titers [50]. Halperin and associates confirmed that anti-*Borrelia* antibodies were not infrequently found in ALS cases that they examined and began to treat with CFT a subset of nine patients at various stages of ALS disease progression who had anti-*Borrelia* antibodies [50]. They found that three patients appeared to improve, three had disease progression and three were apparently unaffected. This initial finding can suggest many possibilities, and some suggest that the time course of the disease has a strong bearing on pathogenesis and on intervention outcomes.

CFT has been reported to have neuroprotective and anti-inflammatory effects. Pre-treatment of CFT attenuates proinflammatory cytokines, such as Nuclear Factor-κB, interferon-γ, and/or tumor necrosis factor-α (TNF-α) and interleukin-1β (IL-1β) in various neurological models of PD [36] neuropathy [37], TBI [38] and cerebral ischemia [51]. Beta lactams are an older class of antibiotics and this is not to suggest that repurposing old drugs for neurodegenerative disease is an efficient and useful approach for reducing the costs of drug development, rather, it demonstrates that we need to encourage investment in basic science and basic discovery and apply those efforts to the most difficult problems facing modern medicine today.

It is important to note that spirochetes, like syphilis, are known as “the great pretenders” as their symptomatology can mimic other diseases. We mentioned cognitive impairment and confusion with Lyme neuroborreliosis, however, spirochetes are not the only microorganisms that hold this title and Lyme consists of other offenders including *Babesia, Bartonella, Richettsia, Mycoplasma*, *Treponema, Leptospira, Coxiella, Ehrlichia chaffeensis, Francisella tularensis,* nematodes, parasites and a host of upwards of 23 pathogens and human viruses, such as human herpes virus 6, which are among the many pathogens that can comprise so-called “Lyme disease” [52]. Lyme disease can affect human nervous tissue as illustrated by the effect on the cardiac neurologic network in patients who develop heart block from Lyme disease [53]. While the association of ALS with Lyme disease is not established, it cannot be ruled out as a causative or contributing factor either [54,55]. However, neurodegenerative diseases are typically multifactorial in their pathobiology. Not all bacterial are either harmful or innocuous and there could be a subset of anaerobes, which we have yet to identify, that specifically contribute to neurologic disease or mimic symptoms of other diseases. Moreover, ALS patients show reduced levels of *Oscillibacter, Anaerostipes*, *Lachnospiraceae* and overgrowth of *Dorea.* While extensive dysbiosis is a hallmark of other neurodegenerative diseases like AD, and PD, overgrown populations nonetheless may contribute significantly to pathogenicity across the gut–brain axis in ALS, causing intestinal inflammation and producing increased gut permeability [56,57].

## 5. Mechanisms of Action for Antibiotics

There are multiple mechanisms for CFT’s neuro-protective action of various neurodegenerative diseases and the common suggestions, including upregulation of Glutamate transporter 1 (GLT-1) expression, attenuation of oxidative stress or neuro-inflammatory process and others already elucidated. CFT obviously has diverse mechanisms of action and has relieved symptoms and the natural course of numerous neurodegenerative diseases. Other than the upregulation of GLT-1 expression, the attenuation of neuroinflammation and of oxidative stress, as well as providing neuroprotection for various neurodegenerative diseases we find it useful in animal models of AD, PD and ALS [56,57,58]. It was found to stabilize synaptic proteins and downregulated the microtubule associated protein tau, prevented cognitive decline and ameliorated pathology and offered neuroprotection in various models of ALS and in clinical trials [59].

So, beta-lactam antibiotics and probiotics could represent not only the effect of antibiotics, but also the helpful nature of commensal anaerobes, which are not necessarily mutually exclusive. Thus, it is imperative that we investigate how specific strains of microbes affect the metabolome composition and homeostasis in neurology disease and identify which species are contributing to ALS and neurodegeneration. Consequently, we can conclude that antibiotic use can create problems for the gut microbiota and correlations with neurological disease and research has demonstrated impaired gut integrity or reduced gut homeostasis plays an active role in ALS and other neurodegenerative diseases.

Rodent models showed that CFT significantly attenuated the inflammatory production of TNF-α and IL-1 β, which mitigated the increased levels of Bcl-2-associated X protein or BAX and forms of caspases, namely, 3 and 9, while amplifying the expression of B-cell leukemia/lymphoma 2 protein, called Bcl2, at the level of apoptosis. Moreover, there could also be a subset of anaerobes, responsive to CFT antibiotics that could contribute to these same symptoms. Generally, it appears that the shared mechanism of actions of CFT in the range of various neurologic disorders is through the upregulation of GLT-1 expression and the reduction of proinflammatory mediators and oxidative stress. However, this requires further investigation in order to verify the suggested and possible other newer neuroprotective mechanisms of CFT for each neurological ailment.

## 6. Mechanisms in ALS Pathogenesis

**Figure 1:**
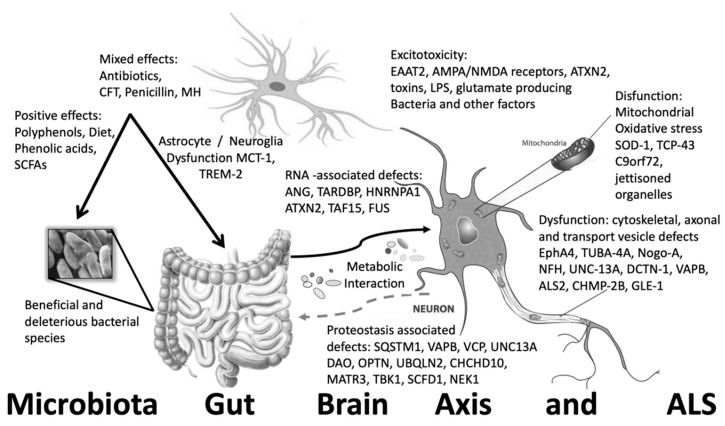
Mechanisms of pathogenesis in amyotrophic lateral sclerosis and neurodegeneration involving key cells, bacteria and systems including genetic, mitochondrial, environmental and epistatic interactions with the gut microbiota and direct bacterial infection mechanisms.

Many common mechanisms are suspected to underlie the etiology of ALS pathogenesis and most neurodegenerative diseases as well. Vulnerable neurons particular to each neurodegeneration process and disease are the key targets. In ALS it is the motor neurons that are vulnerable but in AD it is the pyramidal neurons of the hippocampus and in PD it is the neurons of the substantia nigra. Regardless of the neuronal type or sub-type, each cell and region of the brain or ganglia possess vulnerable neurons, which are the affected cells in each particular disease. Nevertheless, several common assaults are considered to underlie disease pathogenesis. In particular, oxidative stress, neuroinflammation, proteinopathies, excitatory over stimulation, impaired metabolism or bioenergetics, mitochondrial dysfunction, excitotoxic or pathogen exposure and aggregations or inclusions are the most common mechanisms discussed in the literature see Figure 1 [58,60].

These mechanisms are extremely interrelated and are complex with vicious spheres of influence and propagation, which finally lead to cell dysfunction and death. Efforts to curb these stressors and propagators of disease are the subject of many trials and approaches to alleviate symptoms or outright cure these diseases. Unfortunately, time is not on the patient’s side or the time-course of the disease, where the extent of any irreversible damage or virulence factors and paucity of any defense mechanisms are important considerations when discussing any neurodegenerative disease. By focusing on ALS, we also consider commonalities within related brain diseases and this tells us much about disease mechanisms, but this falls short of capturing or elucidating an effective cure. However, when we consider the status of the microbiota-gut in these diseases, we add a layer of biochemistry or protection to help prevent these devastating diseases. For that reason, we suggest we may eventually see progress with a potential cure in sight.

## 7. Oxidative Stress, Excitotoxicity and Inflammation in ALS

One of the older mechanisms underlying the etiology of all neurodegenerative diseases is oxidative stress. Oxidative stress-induced motor neuron death is such a component in ALS pathogenesis. In cases of SOD-1 related familial ALS, antioxidant protein metabolism is already compromised and further oxidative stress can lead to neuronal death through this mechanism. In this regard, it is largely a paucity of endogenous redox defenses either from mutations or with advancing age or because of various disease sequalae, that promotes oxidative stress or prevents its adequate quenching, which underlies this particular stressor. Whether these assaults arise from endogenous or exogenous sources, several antioxidants and free-radical scavengers are proposed to deal with or eliminate the stress. Mechanisms that promote the stress, such as metal-catalyzed oxidation or ionizing radiation, for example, suggest that antioxidants or scavenging approaches will prevent or cure the disease. Although familial ALS does show some trends, most forms of ALS do not have clear mechanisms for disease pathogenesis. However, around 20% of patients do have mutations in the endogenous copper/zinc SOD-1 antioxidant enzyme and patients with FALS tend to develop motor neuron disease that focuses on the lumbar and cervical region of the central nervous system [39]. It is then of great interest to be able to specifically modulate bacterial populations to minimize overgrown and endotoxic and excitotoxic strains or to protect and propagate probiotics that are protective and synthesize key metabolites that would provide colonization resistance to those that contribute to neurodegeneration.

Mutant SOD-1 protein does generate oxygen and nitrogen-free radicals as byproducts of aerobic metabolism and altering peroxidase activity, among other mechanisms [31]. An early study that explored oxidative stress in animal models used transgenic mice for SOD-1 and mouse models lacking SOD-1 did show significant ALS symptoms, but also caused cellular death. Later studies using transgenic mice with mutant G93A-superoxide dismutase-1, which are one of the most commonly used animal ALS models, as is mutant G37R-superoxide dismutase-1 transgenic mice, which show ALS symptoms in significantly greater degrees. We use these models to show the effectiveness of potential therapies. For example, selective metabotropic glutamate 3, or mGlu3, receptor agonist LY379268 enhanced Glial Cell line-derived Neurotrophic Factor (GDNF) and glutamate transporter GLT-1 had a beneficial effect on neurological disability in G93A SOD-I transgenic mice (SOD-1-Tg) mice but had no significant effect on lifespan [61]. Generation of oxygen radicals by altering peroxidase activity and generating lipid peroxidation was shown in a transgenic mouse model study with a mutant form of SOD-1. This group examined the extent to which peroxidase activity damaged spinal cord tissues of transgenic mice compared to controls and showed that spinal cord lipid peroxidation correlates with familial ALS transgenic mice models [31,61]. In cases of SOD-1 related familial ALS, antioxidant protein metabolism is already compromised and further oxidative stress can lead to neuron death. However, studies using transgenic mice for SOD-1 and mouse models lacking SOD-1 reportedly showed significant ALS symptoms, but they caused cellular death [62]. It is known since 1996 that SOD-1 knockout mice develop normally and show few if any sign of neurodegeneration. However, genetic markers suggest we should look at SOD-1 and the other genes correlated with ALS to develop alternative models and explore concomitant microbial involvement in these model, especially during the early pathogenesis of the disease.

It is well-known that abnormally increased levels of glutamate can induce neuronal damage and excitotoxicity that contributes to the pathogenesis of several neurological disorders including Parkinson disease, AD and ALS. As part of a toxicity model of motor neuron disease, impairments with glutamate-transport play a key role and may also operate in ALS pathophysiology. The pre-synaptic glutamate transporter, GLT-1, clears most of the glutamate released in the cortex and hippocampus [63] and enhancing its expression improve neurological outcomes [64]. Moreover, blocking certain Glu receptors or overexpressing specific glutamate transporters involved in terminating glutamatergic transmission are shown to improve various experimental models of neurological disease and may have an antidepressant effect.

The microbiota-gut and antibiotics can have antioxidant effects either from metabolism or through drug interaction. For example, tetracycline derivatives and ceftriaxone protect neurons from oxidative stress and ionizing radiation or act as free radical scavengers [65]. Both beta-lactam antibiotics and Bromocriptine, a dopamine agonist used to treat Parkinson disease, can inhibit oxidative stress-induced cell death and can sustain motor neuron function and modestly prolongs neuronal survival in ALS SOD-1-Tg mice even after disease onset. Although CFT, has different putative mechanisms of action or when co-administered with bromocriptine for ALS treatment. Interestingly, both drugs have similar lactam ring structure, which might serve as a functional, inhibitory or active site for ALS therapy [66].

The correlation between ALS and environmental influences have shown that SOD-1-Tg mice tend to show vivarium-dependent dysbiosis at prodromal stages and abnormal metabolite patterns, when subject to broad spectrum antibiotic treatment [67]. Results from this study show a direct correlation between vivarium with mouse severity, exposed to antibiotic-treated SOD-1-Tg mice and certain bacterial species, particularly *Akkermansia muciniphila*, which alleviates ALS symptoms; *Ruminococcus torques* and *Parabacteroides distasonis*, which escalates ALS symptoms [67]. The mice that were subject to *Akkermansia muciniphila* species additionally showed accumulation of nicotinamide in the CNS. Nicotinamide has been under recent investigation for a correlation to improved ALS symptoms and thus better motor function. In the study, SOD-1-Tg mice were shown to have better ALS motor symptoms. This data was used to identify particular microbiomes and metabolites present during these experimental conditions, in order to create a proposal for future human investigation.

## 8. Mitochondrial Involvement in Oxidative Stress-Induced Neurodegeneration

Mitochondria are key propagators of oxidative stress in many diseases including AD and ALS [20,68,69]. Faulty mitochondria mark other neurodegenerative disorders as well, including amyotrophic lateral sclerosis [69,70]. If we remember the endosymbiont theory, first purported by Lynn Margulis in 1967 [71], the mitochondria are bacterial in origin and their proteins, lipids, and circular DNA are protected by two membranes within the cell from the innate immune system. Further, when damaged they may release bacterial-like macromolecules from this immune privileged space to be immunoreactive and promote inflammation.

Mitochondrial fragments and damaged mitochondria [70], jettisoned by stressed cells and neurons and inflamed astrocytes, in turn, expelled their own mitochondrial fragments [72]. These authors demonstrated that this process is deleterious, and that inhibiting mitochondrial fission prevented gliosis and protected neurons in the Five Alzheimer disease-linked mutations (5xFAD) and an amyloid precursor protein and presenilin-1 transgenic mouse model when P110 treatment was added. This lowered reactive oxygen species, reduced mitochondrial fission and improved ATP production. At the same time P110 treatment reduced proinflammatory cytokine production by one half. This included a reduction in TNF-α and IL-1β. More importantly, microglia that expressed superoxide dismutase with a FALS mutation was improved as was cells treated with the oligomeric Aβ42 form of amyloid [72]. Further, primary astrocyte culture media from rodent cells, demonstrated that astrocytes exported TNF-α and IL-1β and microglia carrying glutamine repeat Q73 or SOD-1 mutations, which can suppress cellular respiration and shorten primary neuron life in culture. If the conditioned media from the astrocyte culture was added to unaffected mitochondria, they became dysfunctional and fragmented. This deterioration was reversed with P110 addition or addition of intact mitochondria, which suggests that mitochondria fission plays a role in this deleterious process and fragmentation of mitochondria causes microglia and astrocytes to release factors that can somehow damage mitochondria in other cells, likely through cytokine stress.

Regardless, there is strong suggestion that leaky gut and LPS or other bacterial component translocation are involved in these inflammatory processes and that increased permeability of the immune privileged barriers plays a role in brain disease and in neurodegeneration [13]. This is evidenced by models of microglia carrying ALS mutations and when models treated with lipopolysaccharide are used to demonstrate shedding of jettisoned damaged cellular components into the media, which suggests that damaged extracellular components were a key source for toxicity [72] and bacterial components may trigger the same inflammatory processes. This is an interesting finding, which further suggests that when damaged mitochondrial debris, LPS or bacterial components are removed from wherever they are found, they could help treat ALS and other neurodegenerative diseases, such as Alzheimer disease by suppressing neuroinflammation among other mechanisms. Further, postmortem brains of ALS white matter lesions suggest it may be attributable to celiac disease [73].

Tanaka and colleagues showed that in late stages of ALS using the late-stage ALS mice, (WN1316) 2-[mesityl (methyl) amino]-N-[4-(pyridin-2-yl)-1H-imidazol-2-yl] acetamide trihydrochloride was observed in conjunction with water solubility and high blood-brain-barrier permeability in selectively suppressing neuronal inflammation and oxidative stress-induced cell death via inflammation [74]. Interestingly, WN1316 increased neuronal apoptosis inhibitory protein and NF-E2-related factor 2, which affected anti-oxidation pathways related to glutathione and protected motor neurons against oxidative stress-induced injury. This data suggests that intravenous glutathione could also help ALS patients and may be dependent on disease status and progression since CFT, when added as an ALS regimen, passes into the CNS of patients with noninflamed meningeal tissue [72]. This suggests that early interventions in ALS may be dependent on the cytokine stress load of ALS patients.

More arguments for oxidative stress and mitochondrial protectants [75,76] in ALS comes from the exploration of mitochondrial superoxide dismutase (mSOD)-induced defective mitochondria, which is associated with the pathogenesis of ALS and other neurodegenerative diseases as evidenced by mitochondrial swelling, giant mitochondria, vacuolization and fragmentation, which are prodromal stages in ALS [75,76] and other neurodegenerative diseases [21].

## 9. Pesticides and Toxic Excitatory and CNS Damaging Compounds

Excitotoxins include glutamate and glutamate analogs, which mimic the action of glutamate at glutamate receptors as well as α-amino-3-hydroxy-5-methylisoxazole-4-propionic acid (AMPA) and N-methyl-D-aspartate (NMDA) receptors. Excitotoxicity can occur from endogenous excitotoxins of which glutamate is a prime example or come from exogenous neurotoxins. As the major excitatory neurotransmitter in the brain and CNS, glutamate concentration can rise sharply in the synaptic cleft to levels approaching 1 millimolar [77,78], which then should sharply fall. If levels cannot be decreased or increases further the neuron can undergo death by apoptotic means [78,79]. This can also occur after brain and spinal cord injury. Eventually, cellular Ca^2+^ influx activates enzymes, phospholipases, endonucleases and calpain, which damage cellular structures.

Neonicotinoids and fipronil for example are widely used insecticides in over one third of the world that act systemically on plants to target organisms with nervous systems. Importantly, fipronil may negatively affect soil microorganism degradation of this pesticide as well and very little is known about the effect these pesticides have on non-target organisms or the ecosystem [80]. Neonicotinoids mimic the action of neurotransmitters, in doing so, they continuously stimulate neurons leading ultimately to death of target invertebrates, while fipronil inhibits neuronal receptors [79,81]. Most insecticides, have lethal and substantial impact on non-target organisms, including those that predate insects and vertebrates. This raises the specter of potential risk to human health and neurologic diseases. Neonicotinoids show agonist activity that generates continuous membrane depolarization, causing major nervous system malfunction. Neonicotinoids have been shown to induce nicotinic acetylcholine receptors (nAChRs), thus synaptic acetylcholine activity is disrupted [79]. Thiamethoxam is a second-generation neonicotinoid, and imidacloprid, which exhibits reduced nAChR activity as an agonist. As compared to fipronil, which causes GABA accumulation via antagonist activity, neonicotinoids exhibit agonist activity. Neonicotinoids also show decreased affinity towards vertebrate receptors, allowing higher selectivity between insects and humans [66]. These studies strongly suggest that imidacloprid, the archetypal Neonicotinoid, is an antagonist at muscle cell neuronal acetylcholine receptors [80,82].

Even though it is a very common neurotransmitter, neuronal hyperexcitation can occur from over-accumulation of the neurotransmitter GABA at the synaptic junction. The sensitivity and selectivity depend on the subunit composition of the human GABAA receptors and contrary to acetylcholine, acetylcholinesterase does not act on nicotine nor imidacloprid, and possibly on the other neonicotinoids, leading to their prolonged action on the nAChRs in insect studies [81,83]. In regards to GABA neurotransmitter production, it is known that these can come from bacterial metabolism and select fruits, functional foods and anthocyanins in particular [83]. These authors show that black carrots fermented with *Lactobacillus plantarum* or *Aspergillus oryzae* prevented cognitive dysfunction by improving hippocampal insulin signaling and we can add that similar approaches were helpful in ALS as well [67]. Li and colleagues identified a high GABA producing species of *Lactobacillus brevis* [84], which can convert sodium glutamate to γ-aminobutyric acid, this could help reduce the glutamate load from dietary sources in the gut. Neuronal hyperexcitation can occur from over-accumulation of the neurotransmitter GABA at the synaptic junction [85]. Here a reference must be provided supporting this affirmation as it seems to contradict fundamental neuroscience principles. Generally, mature GABAergic synapses are inhibitory, as they act to hyperpolarize the postsynaptic neuron, while glutamatergic synapses are excitatory and depolarize, i.e., a nerve firing, at the postsynaptic neuron, while glutamatergic synapses are excitatory and depolarize [85].

In the postsynaptic neuron. glutamate and GABA are the major neurotransmitters in the mammalian brain. Inhibitory GABA and excitatory glutamate together control many processes, including the overall level of brain excitation. Glutamate can function as either an excitatory or inhibitory neurotransmitter. Glutamate is synthesized in the Endoplasmic Reticulum and transported via the Golgi to then emerge in the synaptic cleft surrounded by a vesicular membrane. Once freed from said membrane via exocytosis, glutamate interacts with postsynaptic glutamate receptors to initiate the response cascade. Of subsequent secondary actions, ionotropic receptors may have some correlation to ALS pathogenesis due to the inherent link between these receptors and Calcium ion concentrations. Excess Ca^2+^ has been associated with neuronal death [85].

## 10. Alternative Drugs, Compounds and Antibiotics May Help ALS

Excitotoxins that affect NMDA and AMPA receptors and kainic acid, as well as pathologically high levels of glutamate, can cause excitotoxicity by allowing high levels of divalent calcium cations to enter the cell [84,85]. Minocycline hydrochloride (MH), a semi-synthetic tetracycline derivative and anti-inflammatory drug, exhibits potent neuroprotective activity and inhibits glutamate excitotoxicity in neurons through several proposed mechanisms of action, including direct and indirect inhibitory effects [74]. Minocycline targets multiple secondary injury mechanisms and can potentially target include inflammation, free radicals and oxidative stress, glutamate excitotoxicity, calcium influx, mitochondrial dysfunction, ischemia, hemorrhage, and edema [84]. Shultz and colleagues described the actions of MH, which are anti-inflammatory and neuroprotective through conserved mechanisms that include modulation of phosphoinositide 3-kinase signaling pathways, p38 mitogen-activated protein kinase and by matrix metalloproteinase inhibition. Further, MH can directly inhibit the calcium influx that occurs through NMDA receptors, inhibit mitochondrial calcium uptake, as well as poly (ADP-ribose) polymerase-1 enzymatic activity and toxicity from redox active iron [74]. It can also directly scavenge free radicals, which could be metal catalyzed. Since there are several fronts for MN to affect secondary injury during neurodegenerative mechanisms, MH treatment holds special promise for more than inflammation.

Drugs such as minocycline and ceftriaxone have been tested for therapeutic effects, but the results have been inconclusive, at least under the trial conditions explored. The only approved drugs for the treatment of ALS are currently Riluzole and Edaravone, which only enhance life expectancy by months [43,44,77]. However, alternative mechanisms for drugs, such as Riluzole, Talampanel and Edaravone their effective for the modification of the microbiota [2]. For example, Riluzole protects motor neurons against excitotoxicity-induced degeneration by disrupting glutamatergic transmission and decreasing its concentration and at the same time provides a modest survival benefit in patients [40]. Riluzole and its prodrugs introduced to be more stable in vivo, may improve its use and outcomes. Talampanel, a non-competitive antagonist of α-amino-3-hydroxy- 5-methylisoxazole-4 propionic acid receptors to glutamate-induced excitotoxicity in neurodegenerative diseases and withaferin A, an inhibitor of nuclear factor-kappa B activity, may have beneficial effects on SOD-1 mouse models when administrated at the early stage of the disease [43,86,87]. For example, in a phase two study, ALS functional rating scale, motor deficits were decreased in Talampanel-treated ALS patients [87] and early treatment with withaferin A reduced levels of misfolded superoxide dismutase 1 and extended lifespan in a mouse model of ALS [86].

Bacterial-derived molecules is another interesting approach to neurodegenerative diseases. In that regard, molecular hydrogen specie are of increasing interest since oxidative stress and neuroinflammation cause many neurological disorders. It thus follows that antioxidants and forms of molecular hydrogen (H_2_) could be used for neurological disorders, including cerebrovascular diseases and neurodegenerative diseases [88]. Hydrogen sulfide (H_2_S) and molecular hydrogen may be helpful in alleviating neurologic conditions and could offer an explanation for why bacterial-derived hydrogen could be helpful as a treatment for oxidative stress [88]. Forms of hydrogen are also markers of bacterial metabolism in some cases. Hydrogen in many forms may offer relief or prevention as it functions as both antioxidant and anti-inflammatory agent [88] and has demonstrated efficacy in neurologic diseases, suggesting the clinical potential of H_2_ administration [88]. In animal models and human clinical studies routes for hydrogen administration include drinking H_2_ dissolved in water, injection of H_2_ dissolved in normal saline or inhalation of H_2_ gas. These treatment modalities have offered prevention and mitigation of neurologic diseases [88]. What is not known is the exact mechanism of action for H_2_, but it may be acting through a signaling mechanism. Therefore, further investigation is required to determine the exact molecular targets for H_2_ and its precise mechanisms of action. Nevertheless, hydrogen-rich saline, for example, significantly suppressed reactive oxygen and nitrogen species (ROS, RNS) production, microglial and glial activation and inhibited the release of mitochondrial apoptogenic factors [89]. Subsequently, hydrogen was shown to suppress 3-nitrotyrosine and activation of caspase-3, as well as decrease protein carbonyl levels and the formation of damaging peroxynitrite and malondialdehyde [90].

Finally, vitamin D use in rodent cortical neurons protected against glutamate excitotoxicity and kainic acid [89,91] and high-dosing improved motor performance and grip endurance in G93A mice [89,92]. Clinically, vitamin D3 reduced the decline in ALSFRS-R score [92]. Can we recommend high dose vitamin D? Not necessarily, but we should examine the role and prevalence of ALS in countries above the 43rd parallel in the northern hemisphere and opposing sites in the southern hemisphere for a link to sunlight exposure or lack thereof.

## 11. Excitotoxic Agents, Timing and Alternative Mechanisms for Antibiotics

Alternatives to beta lactam mechanisms of action exist for ALS. Many neurological pathologies are being tightly linked to metabolic pathways that lead to excitotoxicity. Excitotoxicity to nerve and brain cells can be damaged or killed by excessive overstimulation. Excitotoxicity can be defined as the state in which neurons exhibit extreme activation of amino acid receptors such as aspartate or glutamate, neurotransmitters, which are produced in the endoplasmic reticulum transported via the Golgi to synaptic vesicles and then to gap junctions [85,93]. This excitotoxicity can come from neurotransmitters, such as glutamate, aspartate and similar substances. Accumulation of glutamate and aspartate in the extracellular fluid follows ischemic brain events, causing cell death. Select antagonists can halt the neurotoxicity to receptors during the so-called glutamatergic storm.

Glutamate excitotoxicity is implicated as one factor contributing to a plethora of different disorders such as trauma, seizures, and neurodegenerative diseases. When examining postsynaptic glutamate receptors, it can be observed that the majority consist of ionotropic and metabotropic receptors, which tend to be G-protein linked. When receptors for AMPA and NMDA are overactivated, neuronal cellular damage can occur at relatively low levels of glutamate, reportedly in the 10 micromolar (μM) range [29,76,91]. Perhaps this may occur even at sub-micromolar levels. Once activated, this leads to the ultimate mechanism used to release Calcium for the sarcoplasmic reticulum which facilitates membrane depolarization. Overstimulation via Calcium ions can cause associated neuronal death. In healthy neurons, homeostatic calcium levels are kept at very low concentrations. If we compound the damage from previous mechanisms with that from over-introduction of glutamate, aspartate or excitotoxic pesticides, and their respective agonists, we now describe an extremely potent mechanism for neurodegeneration.

Moreover, excitotoxicity through this process also leads to uncontrolled Ca2+ ion influx and the pathophysiology described, which is another component to excitotoxicity not often discussed. Calcium levels are maintained by antiporter transport of calcium ions to extracellular spaces and cell membranes. However, in the excitotoxic cell, calcium concentrations in excess over activate enzymes, such as endonucleases, proteases, kinases and phosphatases, which differ in their roles in cell homeostasis. Once activated, these enzymes contribute to cell death or apoptosis in the affected cell due to mass enzyme over activation. ALS does share features with other neurodegenerative diseases and all of them may have a bacterial component to their pathogenesis. Therefore, we cannot exclude a role for the microbiota in this neurodegenerative disease as well.

ALS and other neurodegeneration also compromises the proteostasis, in the brain and elsewhere, in a manner that parallels impaired metabolism and signal molecule synthesis in the gut through dysbiosis. Because the majority of bodily genes are contained in the gut bacterial genomes, it is then important to be able to modulate and engineer microbial metabolism in compromised and disease-state guts to restore microbiome proteostasis either by enhancing expression of probiotic genes, or suppression of endotoxic metabolites such as β-methylamino-L-alanine (BMAA) [82,94] or triglyceride metabolism and lipoprotein lipase. Alzheimer animal model studies using the vervet *Chlorocebus sabaeus* with an apoE4 homozygous background, developed hallmark AD pathology including neurofibrillary tangle and Aβ plaque accumulation after orally administered β-methylamino-L-alanine [82,95]. Further, in ALS and PD patients, β-methylamino-L-alanine has been detected in post-mortem brain tissue. 

Beyond excitotoxicity is the suggestion that a subset of microbes, perhaps a cyanobacteria species [96,97], may influence ALS pathogenesis. There are reports on *Leishmania major*, which up-regulate pro-inflammatory mediators and in macrophages, already infected with ALS, suggest innate immunity against Leishmania infection is involved in ALS. In that regard, one suspect amino acid derivative, BMAA, was first associated with Parkinsonism dementia and amyotrophic lateral sclerosis as a complex syndrome and identified as a neurotoxic compound in the Chamorro people of Guam [95,97,98,99]. The compound was identified as bacterial-derived and is attributed to oxidative stress from nitrogen species [96]. We know from work with cerebrovascular diseases that oxidative stress in the cardiovascular system, brain and CNS, including brain microvasculature and parenchyma, results in an accumulation of damaging ROS and RNS [99,100]. These compounds promote leukocyte and other immune cell adhesion and increase endothelial and other cellular compartment permeability resulting in chronic injury stimulus and a cascade of downstream events. One in particular is mitochondrial damage, which feeds forward oxidative stress. Other cascades include imbalance in activity of vasoactive substances, such as different isoforms of nitric oxide synthase, endothelin-1, various oxidative stress markers and mitochondrial DNA damage and changes in mitochondrial enzymes in the vascular wall and in brain parenchymal cells [99,100].

Excitotoxicity was one process suspected in ALS pathogenesis, whereby nerve cells are disrupted and poisoned by excessive stimulation from select neurotransmitters like glutamate and Aspartate. Glutamate hyperexcitability and excitotoxicity may contribute to the pathophysiology of ALS and it is believed that glutamate excitotoxicity-induced motor neuron death is involved in the pathogenesis of ALS, which if reduced, glutamate level targeting is one therapeutic approach for ALS [45]. In support of this notion are ALS animal model studies that show decreased excitatory amino acid transporter 2 (EAAT2) overexpression delays onset and prolongs survival, and that CFT increases EAAT2 activity in rodent brains. Conversely, if compromised, EAAT2 does not take up free glutamate from the extracellular space and triggers cellular excitotoxicity, characterized by of glutamate receptor overactivation in postsynaptic neurons.

Ceftriaxone, protects neurons against increase glutamate transporter gene expression, inhibits apoptosis, glutamate neurotoxicity and extend survival time [95]. CFT phase one to three clinical studies occurred in three stages for ALS and were undertaken with good results but time dependent efficacy was noted [51]. Ceftriaxone affects the glia, which is one key component in ALS pathogenesis, which increases the expression of EAAT2 proteins in glial cells. Further, EAAT2/GLT-1 overexpression delayed onset and prolonged survival in ALS mice. Thus, targets that upregulate EAAT2 may be neuroprotective in ALS [100,101,102]. Further, mouse models of Multiple Sclerosis show that reduced activity of EAATs, when coupled with immune cell response to rapid glutamate level increases, leads to degeneration of the CNS and to motor impairment that is signaled by activated T cells. Activated T-lymphocytes do cross the cerebral vascular endothelium to impair blood-brain barrier permeability, promote downstream inflammatory cell migration and affect brain and spinal cord tissue [103]. In that regard, animal models of experimental autoimmune encephalomyelitis show striking clinical improvements in animal subjects, without influencing EAAT2 expression levels or glutamate uptake. This study shows that glutamate transporters are not the chief receptor in the drug mechanism of CFT, as previously suggested but they play a role in the proinflammatory response which is crucial to MS pathogenesis through modulation of myelin-antigen receptors crucial to the permeability of immune cells and retards the migration of T-Cells and other cells across membrane barriers into the CNS.

Ceftriaxone clinical trials for ALS failed to show efficacy in alleviating neurological symptoms in ALS patients. Which is why timing and staging of the disease is important with CFT dosing and perhaps why the trials failed. During stages one and two, by the revised ALS functional rating scale (ALSFRS-R) decline was slower in participants taking 4 grams of ceftriaxone versus a placebo. Despite promising stage-2 efficacy data, the stage-3 CFT in ALS study failed to show clinical efficacy or stage three survival. In phase two, the pharmacokinetics of CFT in plasma and cerebrospinal fluid were planned and for clinical trials in ALS. Ceftriaxone does not significantly increase the survival time or significantly decrease the rate of decline in function for subjects with ALS. Taken together, this suggests that CFT is likely to only be beneficial at early stages of the disease progression and anecdotal evidence from our private practice suggests the same is true [61]. We should be discouraged by trial outcomes, but approach the problem from different angles and some should focus on the microbiota–gut–brain axis and bacteria and host co-metabolism.

## 12. Microglial and Astrocyte Activation in ALS and other Neurodegenerative Diseases

Multiple factors contribute to the neuroinflammation consistent with infection, including microglial, inflammasome, complement activation, cytokine alteration and cytokine stress [15,104]. These processes result in progressive motor neuron degeneration and eventually to neuronal death in ALS. Alzheimer disease is no exception and patients exhibit inflammation and infectious agents are found in the brain and somehow contribute to AD pathogenesis. Moreover, amyloid-beta has been characterized as an antimicrobial peptide, which suggests possible infectious causes for AD and other neurodegeneration [105,106,107,108]. However, causal evidence has not been clearly established. CSF is considered a “window” into brain infection, and clinical AD patient CSF provides insight into the neuropathogenesis of infectious agents [108].

Other immune cell and glia-based mechanisms include aberrant astrocyte function, which are normally neuronal protective in stroke, spinal cord injury and other diseases, but may lead to PD and other neurodegenerative diseases [10,109]. Astrocytes, in the presence of certain inflammatory molecules produced by microglia, appear to become degenerate and aberrant immune cells, which are called neurotoxic reactive astrocytes. Activated microglia, induce the change and increase in abundance. Tissue samples from patients from brain injuries and major neurological disorders, including, Alzheimer disease, Parkinson disease, multiple sclerosis and likely ALS are suspected to have these changes. In fact, researchers have found that bacteria-derived LPS induced this conversion of normal astrocytes into the inflammation promoting A1 type astrocytes. There is microglia activation by LPS, which could induce A1 type astrocyte transformation through glial inflammatory molecules. In rodent studies, molecules produced by microglia were identified and changed astrocyte function and behavior when exposed to LPS, complement factor C1q, TNF-α and Interleukin-1 α (IL-1 α). While each of these substances could partially induce A1 astrocytes, when combined, they promote the complete A1 astrocyte transformation.

Astrocytes normally feed and protect neurons but formed A1 astrocytes actually kill them. Vulnerable neurons and dopaminergic neurons involved in PD die as A1 astrocytes produce increasing amounts toxic cytokines. In the presence of normal astrocytes, retina cells grow and thrive. However, retina cells, whose survival in culture depends on astrocyte contact and support, die in the presence of A1 astrocytes. In mice where the optic nerves were damaged, increased A1 astrocytes were found, which could be inhibited using antibodies to TNF- α, IL-1- α, and C1q. Brain samples from AD, PD, Huntington disease, ALS and multiple sclerosis patients also found high numbers of A1 astrocytes accumulated in the affected regions in each of the respective diseases. Currently, efforts are ongoing to identify and characterize which molecules from A1 astrocytes cause the observed effect in neurons.

Examining associations between ALS and various cell types of the CNS with proteinopathies and the 43kDa transcriptional repressor DNA-binding protein-43 (TDP-43), demonstrates how microglia lead to the progression of ALS. This study examined microglial activity throughout the neurodegeneration of human TDP-43 and it was noted that regardless of severe neurodegeneration occurring in particularly the spinal cord, microglia remained unaffected in inflammatory response and indicated a neuroprotective role for microglia in the CNS and ALS [109]. In microglia activation, TAR-DNA binding protein-43, which is vital in mRNA development, was found to increase the release of neuroinflammatory mediators—Nitric Oxide2, TNF-α, and IL-1β. When in conjunction with cytokines and ROS, elevated TDP-43 produces increased microglial toxicity [110]. Following into a commonly used parallel ALS disease model, SOD-1 mice with both the active and inactive mutants show a progressive neurodegenerative phenotype [110]. Specifically, mSOD-1 generates an altered tertiary structure, which then differentiates the resulting microglial response [111].

In recent years, neuroinflammation and various inflammatory markers have become extremely prevalent in regard to the mechanisms and pathologies of AD and ALS. Neuroinflammation is primarily mediated by two types of cells—microglia and astrocytes. Under appropriate activation conditions surrounding CNS injury or damage, microgliosis and astrogliosis responses are elicited, resulting in increased levels of both microglia and astrocytes at CNS insult sites [112]. Two of the identification markers for activated microglia and astrocytes are glial fibrillary acidic protein, or GFAP, and calcium-binding protein, respectively [113,114]. According to Zhang and colleagues microgliosis and astrogliosis responses likely follow a cascade pattern in which products, such as cytokines, released from microglia induce astrogliosis development [113].

The role of microglia and astrocytes is unique in that they engage in dual opposing roles— neuroprotective and neurotoxic—during the progression of ALS [110]. The microglia neuroprotective properties are evident in the early stages of ALS, while the neurotoxic effects begin to present themselves as CNS damage increases and ALS progresses into the late stages. The neuroprotective phenotype encapsulates tissue repair and anti-inflammation, among additional neuron support characteristics [32,110]. In late-stage ALS, the neurotoxic role of microglia produces significant inflammation, cytokine stress and reactive oxygen species production [32,110]. Cytokines and free radicals are evidenced to be linked to mSOD-1, as discussed earlier. Additionally, mSOD-1 has been identified to negatively impact neurotrophic factor expression, which are vital for proliferation and growth of cells and neurons [111]. As neurotrophic factors are reduced within the CNS, cytokines can garner a stronger inflammatory response, which can result in degeneration of motor neurons [111].

## 13. Neurotrophic and Other Factors Involved in ALS

ALS is multifactorial and we should offer explanations for neuronal loss involving trophic support from neurotrophic factors, such as brain-derived neurotrophic factor (BDNF) and glial cell line-derived neurotrophic factor since this is one characteristic of ALS pathology. Several neurotrophic factors have been suggested as candidate agents for ALS therapy and are demonstrated to promote neuroprotective and neuro-regenerative effects in mouse models of ALS [66]. The expression of GDNF maintained neuronal axonal projections, extended animal lifespan, prevented motor neuron atrophy and retarded the progression of disease in ALS transgenic mice [114]. How the microbiota help maintain or disrupt this mechanism is not known.

Polyphenols and flavonoids show promise in treating neureases [115]. For example, the flavonoid 7,8-dihydroxyflavone, a selective and potent small molecule tyrosine kinase receptor B agonist, was shown to mimic the effects of BDNF, significantly improving motor deficits, preserving spinal motor neurons count and dendritic spines in SOD-1(G93A) mice [116]. The role of the microbiota and the protective nature of polyphenols cannot be overestimated and may be helpful in curbing the role of pathogens in ALS and neurodegenerative diseases. Case in point, polyphenol-rich extracts from *Clostridium nucifera* [117] when used against the protozoan parasite *Leishmania* show pharmacological and anti-leishmanial activity against *Leishmania amazonensis*, suggesting that polyphenolic-rich extract constitutes important advantages for the development of new anti-leishmanial therapies [118]. A limited number of studies have demonstrated the protective activity of polyphenols and phenolic acids like chlorogenic acid, which is present in coffee and was shown to protect cortical primary neurons against glutamate neurotoxicity and thus potentially for ischemic stroke injury [7,91,115].

Some progress has been made in understanding neurodegenerative diseases by exploring extracellular RNAs and DNA/RNA, mRNA, micro RNA, circular RNA and binding proteins, such as TDP-43, which are dysregulated and thus serve as biomarkers of SALS and other neurodegenerative diseases. Exploring extracellular proteins and RNAs in disease suggest they play a role in cellular processes surrounding neurodegenerative diseases and may present a future treatment target for ALS [119].

Nootropics, stimulants like caffeine are suspected to have an excitatory role in or even contribute to excitotoxicity and have an association with neurological pathologies. However, for ALS and AD, there does not seem to be much data to illustrate how stimulants, such as from caffeine consumption relates to the progression of the disease and in fact stimulants even may be protective [120]. Moreover, coffee tea and caffeinated products were explored in a large study in which results were compiled from eight different studies totaling 351,565 participants. Using a Cox regression analysis and confidence intervals, the relationship between caffeine consumption to ALS mortality was tracked. After sufficient time had passed, the results were then gathered and analyzed, and the authors found that 545 participants had passed away from ALS. The results of this study did not suggest there was any significant association between caffeine and ALS pathology to be protective or adverse [121].

## 14. Engineering Approaches to Combat Neurodegeneration and ALS

Clustered regularly interspaced short palindrome repeats (CRISPR) has applications for microbiome, neuroimmune system engineering and therapeutics. CRISPR-CAS is most recognized for its use in permanent heritable genetic changes, but the engineering of therapeutic probiotics is novel and there is interest for it as a transient and nonheritable approach to microbial manipulation. More permanent upregulation of protein expression can be achieved by CRISPR modification of promoter regions to act as inducers, enhancers, i.e., increase affinity for RNA polymerase or for the removal of repressor effects [9]. The use of CRISPR to optimize promotor affinity is sequence specific and targets individual genes of interest while minimizing off target sequence changes.

Future probiotics may be modified to express antimicrobial peptides, therapeutic signal molecules, repair defective enzymes, block bacterial exopolysaccharides [122] or for creating a host of other metabolites and nutritional compounds that may be missing from modern diets [91]. CRISPR is a bacterial immune system for defense against viral and bacteriophage infection, wherein sequences of phage DNA are archived after infection. These sequences are used to detect and cleave viral DNA in subsequent infections using CRISPR associated protein enzymes (CAS) [123]. This recognition and restriction system has been adapted as a robust genetic engineering tool with the ability to identify, cleave, and modify target DNA sequences with greater ease than previous techniques, namely Zinc-finger nucleases and transcription activator-like effector nucleases [124]. As a tool for further modification through the inactivation of the CAS nuclease activity, the so-called dead CAS (dCAS), is a means for affecting genetic transcription through local binding in concert with transcriptional activators or repressors to up or down regulate genetic expression [17]. The dead CAS motif can be used to effect noninheritable transcriptional changes in organisms.

Neurodegenerative diseases have been shown to be difficult to treat with traditional pharmaceuticals and traditional medicine. The difficulty attributed to neurodegeneration is characterized by complex genetic, environmental, epistatic and epigenetic factors, especially in ALS, which make it challenging to target individual risk factors for treatment. Engineering and modification of the microbiome targeting the bidirectional gut–brain axis may be a viable and more broad approach to affecting neurological changes and ameliorating the intestinal effects of microbiome dysbiosis concomitant with neurodegeneration [62]. CRISPR engineering can be used to modulate microbial populations through selective antimicrobial targeting, bacterial metabolism engineering through up and downregulation of genes and insertion of new or beneficial genes.

Traditional antibiotic treatments are often nonspecific and some target intestinal bacteria broadly, which potentially causes indiscriminate or bystander damage and potentially fosters dysbiosis. This happens when populations of desirable commensal bacteria are reduced allowing overgrowth of potentially damaging strains, which we call loss of colonization resistance. Furthermore, the complex communities of diverse bacteria in the gut present challenges to identification and quantification. However, high throughput sequencing techniques across the 16s rRNA region allow large communities of microbes to be sequenced simultaneously for subsequent identification and cataloging. The 16S ribosomal RNA marker is the preferred approach to genetically fingerprinting bacteria; the 16S region is highly conserved and hypervariable between strains [41]. CRISPR-CAS9 has instead been shown to discriminate on a sequence-specific level and delivered via phage to target antibiotic resistance [41,60]. Using phage delivered CRISPR antimicrobials, the specific 16s sequences of Gram-negative bacteria can be targeted. Additionally, the use of high throughput sequencing makes analysis possible of individual patient microbiomes so that these CRISPR antimicrobials can be tailored to treat patient specific dysbiosis.

An interest in disease CRISPR technology can be applied to create permanent heritable genetic changes through CAS9 editing, or more temporary modulations in expression through the use of CRISPR activation (CRISPRa) or inactivation (CRISPRi) using dCAS to bind close to promoter regions in genes of interest. Bound dCAS, conjugated with transcriptional activators, has been used to target transcription factors such as p65, HSF1 and MyoD and increase expression and could be one approach to immunomodulation of the neuroimmune system [60]. CRISPR-Cas9 antimicrobials and the CRISPRa motif can be used to target microbial genes encoding for compromised proteins and metabolites and boost antioxidant activity through the overexpression of gut metabolites, such indole-3-propanol, short chain fatty acids and perhaps GABA [17,91] or glutamate modifying enzymes, for example. In contrast, CRISPR-interference can be used to nonlethally suppress or silence expression of bacterial endotoxins and inflammatory signaling molecules. This approach can more gingerly approach microbiome dysbiosis in a way that does not drastically change bacterial populations, while at the same time still achieves therapeutic effects.

## 15. Conclusions

Many mechanistic studies mentioned above demonstrated effective means to modulate disease progression and survival in ALS mouse models. Several of them are currently considered as potential agents in ALS treatment [66]. However, seldom do promising treatments in animal models translate to effective measures, particularly in ALS patients. However, we know that the timing of these treatments is important during the course of the disease. Moreover, newer concepts, including the role of the microbiota–gut–brain axis in neurodegenerative diseases, limiting overuse of select antibiotics and repurposing older drugs should be considered. Translational failures might be explained in part by the difficulty in getting drugs past the BBB and administration timing, such as before or during early onset of disease, seems to obtain more positive results in animal models at least. Why this cannot be recapitulated in humans is a research and early diagnostic question.

Obstacles to ALS progress are it is rarity and its disease pathogenesis and progression is largely unknown, as are the sites of mechanistic action, which remain unclear or underexplored today. For example, some questions arise such as (1) do treatments directly or partly affect neurons themselves or (2) do they affect the microbes of the gut? (3) Are there subsets of particularly pathogenic organisms that play major roles and if so (4) are their populations transient and (5) have they even yet been classified or cultured? Particularly difficult with trials involving deadly diseases, like ALS are stratifying and grouping issues, where assignments into control or placebo groups becomes unacceptable to patients who want the treatment and not a placebo making comparative results difficult to get for this rare condition. Further, if studies are utilized, they may be best presented as “head to head comparisons” with similar treatment standards and approaches. Importantly, mechanistic studies should focus on understanding precise mechanisms of action for the putative theories suggested to underly human ALS pathobiology. Moreover, identifying and highlighting biochemical biomarkers for this disease are needed as is optimizing characterizing antibiotics or drugs as they impact the eubiota, which may be involved during the course of the disease.

Understanding the extent to which immune and CNS cell types are involved and active in the progression of ALS is not well characterized. The association between ALS and immune cells, such as microglia and regulatory T-lymphocytes, have been of particular interest. Previous studies have illustrated that there appears to be a correlation between gut dysbiosis, inflammation and early pre-onset ALS. Furthermore, we know that the gut microbiota directly influences T-regulatory immune cells by influencing cell numbers and immune responses. Thus, increasing basic science funding in the area of neuroimmunology and inflammation pathway elucidation and remediation could prove rewarding for patients. This correlation indicated a potential link between gut microbiota and ALS pathogenesis [48] and because beta lactam antibiotics have been helpful, it is more imperative we work on this front to provide effective treatments, if only for some. fecal transplantation has been documented to help restore gut integrity and natural immune response conditions and studies propose the use of fecal transplantation as a means of therapeutic relief for the gut microbiota in ALS, PD and AD. It is plausible that through the use of fecal transplant, we will see an increase in T-reg cell counts as well as improved responses to ALS [48] in early case intervention.

Glutamate excitotoxicity, if a major ALS contributor, is not mutually exclusive with a hypothesis involving a subspecies of ALS causing bacteria because there are species of microbes that are known to overproduce glutamate. Glutamate production can occur by many organisms, including *Brevibacterium lactofermentum* and glutamine from *Corynebacterium glutamicum* [125] and the key enzymes that are involved in synthesis are largely those of ammonium assimilation, which include nicotinamide adenine dinucleotide phosphate-glutamate dehydrogenase, glutamine synthetase, glutamate synthase Amino Transferase (GOGAT) and alanine dehydrogenase. The two enzymes in enteric organisms, which mainly account for ammonia incorporation are glutamine synthetase and GOGAT. They constitute the system where glutamine synthetase converts glutamate and ammonia to glutamine and GOGAT converts glutamine and alpha-ketoglutarate to two moles of glutamate [7,93]. Thus, these bacteria could contribute to the glutamate storm and other bacteria that metabolized or fermented glutamate to acetate, butyrate, carbon dioxide, and ammonia, could help diminish overproduction of glutamate, namely, through enzyme pathways methyl-aspartate and the hydroxy-glutarate, which are used by *Clostridium tetanomorphum* and *Peptococcus aerogenes*, respectively [7,25]. Moreover, glutamate utilization is important for establishment of *Clostridium difficile* in the animal gut [92]. Amino acid metabolism by *Clostridium sticklandii*, particularly glutamate, can occur with the following enzymes: Glutamate dehydrogenase, Glutaconate CoA-transferase, Glutaconyl-CoA decarboxylase, Short-chain acyl-CoA dehydrogenase, Acetate CoA-transferase and help utilize excess glutamate [91].

While the glutamate excitotoxicity theory of ALS is not clearly established, what we do know is that microbes somehow contribute to ALS or possibly to excitotoxicity and select antibiotics have shown beneficial effect while others appear to be deleterious. For example, Ciprofloxacin, which is an inhibitor of transcription and bacterial topoisomerase and DNA gyrase was shown to inhibit growth of *Clostridium glutamicum* with concomitant production and excretion of glutamate^119^. Enzyme assays showed Ciprofloxacin addition decreased 2-oxoglutarate dehydrogenase activity even though other elicitors of glutamate production were absent from the medium [7]. Taken together, it is likely that microbes contribute to glutamate toxicity, where antibiotics could play a role in ALS pathogenesis and commensal probiotics or select antibiotics could benefit the disease in early stages. Conversely, there may be a subset of deleterious microbes that contribute to or cause the glutamate storm and the same subset may be involved in neurodegeneration in multiple neurologic diseases, including ALS. While the microbiota–gut–brain axis holds promise for disease treatment and prevention, we still have far to go to elucidate what is most important about them where neurologic diseases are concerned. Time is not on the side of the patient, so to get there we need more than ideas and reviews, we need funding and only then can we explore the possibilities, if we are to treat, cure or eradicate refractory and terribly devastating neurodegenerative diseases.

## Abbreviations

MGB	microbiota–gut–brain axis
AD	Alzheimer disease
ALS	Amyotrophic Lateral Sclerosis
GM	gut microbiota
GABA	γ-aminobutyric acid
PD	Parkinson disease
LPS	lipopolysaccharide
Ca^2+^	calcium
BBB	blood brain barriers
Aβ	amyloid-beta
SCFA	short chain fatty acids
ALSFRS-R	ALS Functional Rating Scale-Revised
SALS	Sporadic ALS
FALS	Familial ALS
SOD-1	Super Oxide Dismutase-1
TAR	Trans-activation response
CFT/CEF	Ceftriaxone
TBI	traumatic brain injury
TNF-α	tumor necrosis factor-α
IL-1 α	Interleukin-1 α
IL-1 β	Interleukin-1β
5xFAD	Five Alzheimer disease-linked mutations
GLT-1	Glutamate transporter 1
CNS	central nervous system
GDNF	Glial Cell line-derived Neurotrophic Factor
SOD-1-Tg	SOD-I transgenic
mSOD	mitochondrial superoxide dismutase
AMPA	α-amino-3-hydroxy-5-methylisoxazole-4-propionic acid
NMDA	N-methyl-D-aspartate
nAChRs	induce nicotinic acetylcholine receptors
MH.	Minocycline hydrochloride
H2	molecular hydrogen
H2S	Hydrogen sulfide
ROS/RNS	reactive oxygen and nitrogen species
μM	micromolar
BMAA	β-methylamino-L-alanine
EAAT2	excitatory amino acid transporter 2
TDP-43	transcriptional repressor DNA-binding protein-43
BDNF	brain-derived neurotrophic factor
CRISPR	Clustered regularly interspaced short palindrome repeats
CAS	CRISPR associated protein enzymes
dCAS	dead CAS
CRISPRa	CRISPR activation
CRISPRi	CRISPR inactivation
GOGAT	glutamine synthetase, glutamate synthase Amino Transferase
C9ORF72	chromosome 9 open reading frame

## References

[1] Hill J.M., Clement C., Pogue A.I., Bhattacharjee S., Zhao Y., Lukiw W.J. (2014). Pathogenic microbes, the microbiome, and Alzheimer’s disease (AD). Front. Aging Neurosci..

[2] Sasmita A.O. (2019). Modification of the gut microbiome to combat neurodegeneration. Rev. Neurosci..

[3] Obrenovich M.E., Tima M., Polinkovsky A., Zhang R., Emancipator S.N., Donskey C.J. (2017). Targeted metabolomics analysis identifies intestinal microbiota-derived urinary biomarkers of colonization resistance in antibiotic-treated mice. Antimicrob. Agents Chemother..

[4] Wong C.B., Kobayashi Y., Xiao J.Z. (2018). Probiotics for preventing cognitive impairment in alzheimer’s disease. Gut Microbiota-Brain Axis.

[5] Govindpani K., Calvo-Flores Guzmán B., Vinnakota C., Waldvogel H.J., Faull R.L., Kwakowsky A. (2017). Towards a better understanding of gabaergic remodeling in alzheimer’s disease. Int. J. Mol. Sci..

[6] Barrett E., Ross R.P., O’Toole P.W., Fitzgerald G.F., Stanton C. (2012). Gamma-Aminobutyric acid production by culturable bacteria from the human intestine. J. Appl. Microbiol..

[7] Girinathan B.P., Braun S., Sirigireddy A.R., Espinola-Lopez J., Govind R. (2016). Correction: Importance of glutamate dehydrogenase (gdh) in clostridium difficile colonization in vivo. PLoS ONE.

[8] Li T., Zhang S. (2016). Microgliosis in the injured brain: Infiltrating cells and reactive microglia both play a role. Neuroscientist.

[9] Uddin M.S. (2019). Nootropic and anti-alzheimer actions of medicinal plants: Molecular insight into therapeutic potential to alleviate alzheimer neuropathology. Mol. Neurobiol..

[10] Fernandes J. (2017). Aberrant astrocytes may lead to parkinson’s, other neurodegenerative diseases. Nature.

[11] Venegas C., Kumar S., Franklin B.S., Dierkes T., Brinkschulte R., Tejera D., Vieira-Saecker A., Schwartz S., Santarelli F., Kummer M.P. (2017). Microglia-derived asc specks cross-seed amyloid-β in alzheimer’s disease. Nature.

[12] Di Domenico E.G., Cavallo I., Bordignon V., D’Agosto G., Pontone M., Trento E., Gallo M.T., Prignano G., Pimpinelli F., Toma T. (2018). The emerging role of microbial biofilm in lyme neuroborreliosis. Front. Neurol..

[13] Obrenovich M.E.M. (2018). Leaky gut, leaky brain?. Microorganisms.

[14] Coureuil M., Lécuyer H., Bourdoulous S., Nassif X. (2017). A journey into the brain: Insight into how bacterial pathogens cross blood-brain barriers. Nat. Rev. Microbiol..

[15] Poole S., Singhrao S.K., Chukkapalli S., Rivera M., Velsko I., Kesavalu L., Crean S. (2015). Active invasion of porphyromonas gingivalis and infection-induced complement activation in apoe-/- mice brains. J. Alzheimers Dis..

[16] Spangenberg E.E., Lee R.J., Najafi A.R., Rice R.A., Elmore M.R., Blurton-Jones M., West B.L., Green K.N. (2016). Eliminating microglia in alzheimer’s mice prevents neuronal loss without modulating amyloid-β pathology. Brain.

[17] Barrangou R., Fremaux C., Deveau H., Richards M., Boyaval P., Moineau S., Romero D.A., Horvath P. (2007). Crispr provides acquired resistance against viruses in prokaryotes. Science.

[18] Raina A.K., Sayre L.M., Atwood C.S., Rottkamp C.A., Hochman A., Zhu X., Obrenovich M.E., Shimohama S., Nunomura A., Takeda A. (2002). Apoptotic and oxidative indicators in Alzheimer disease. Apoptosis Techniques and Protocols.

[19] Obrenovich M.E., Raina A.K., Ogawa O., Atwood C.S., Morelli L., Smith M.A. (2003). Alzheimer disease—A new beginning, or a final exit. Cell-Cycle Mechanisms in Neuronal Death.

[20] Obrenovich M.E., Joseph J.A., Atwood C.S., Perry G., Smith M.A. (2002). Amyloid-β: A (life) Preserver for the Brain. Neurobiol. Aging.

[21] Aliev G., Palacios H.H., Gasimov E., Obrenovich M.E., Morales L., Leszek J., Bragin V., Solís Herrera A., Gokhman D. (2010). Oxidative stress induced mitochondrial failure and vascular hypoperfusion as a key initiator for the development of alzheimer disease. Pharmaceuticals.

[22] Obrenovich M.E., Smith M.A., Siedlak S.L., Chen S.G., de la Torre J.C., Perry G., Aliev G. (2006). Overexpression of grk2 in alzheimer disease and in a chronic hypoperfusion rat model is an early marker of brain mitochondrial lesions. Neurotox. Res..

[23] Obrenovich M.E., Mana T.S.C., Rai H., Shola D., Sass C., McCloskey B., Levison B.S. (2017). Recent findings within the microbiota-gut-brain-endocrine metabolic interactome. Pathol. Lab. Med. Int..

[24] Jones L., Kumar J., Mistry A., Sankar Chittoor Mana T., Perry G., Reddy V.P., Obrenovich M. (2019). The transformative possibilities of the microbiota and mycobiota for health, disease, aging, and technological innovation. Biomedicines.

[25] Fang X. (2016). Potential role of gut microbiota and tissue barriers in parkinson’s disease and amyotrophic lateral sclerosis. Int. J. Neurosci..

[26] Tefera T.W., Wong Y., Barkl-Luke M.E., Ngo S.T., Thomas N.K., McDonald T.S., Borges K. (2016). Triheptanoin protects motor neurons and delays the onset of motor symptoms in a mouse model of amyotrophic lateral sclerosis. PLoS ONE..

[27] Buckel W., Barker H.A. (1974). Two pathways of glutamate fermentation by anaerobic bacteria. J. Bacteriol..

[28] Louis P., Flint H.J. (2017). Formation of propionate and butyrate by the human colonic microbiota. Environ. Microbiol..

[29] Ansell J., Parakar S., Paturi G., Rosendale D., Blatchford P. (2013). Modification of the colonic microbiota. Advances in Food and Nutrition Research.

[30] Roychowdhury S., Cadnum J., Glueck B., Obrenovich M., Donskey C., Cresci G.A.M. (2018). Faecalibacterium prausnitzii and a prebiotic protect intestinal health in a mouse model of antibiotic and clostridium difficile exposure. J. Parenter. Enter. Nutr..

[31] Obrenovich M., Siddiqui B., McCloskey B., Reddy V.P. (2020). The microbiota-gut-brain axis heart shunt part i: The french paradox, heart disease and the microbiota. Microorganisms.

[32] Hall E.D., Andrus P.K., Oostveen J.A., Fleck T.J., Gurney M.E. (1998). Relationship of oxygen radical-induced lipid peroxidative damage to disease onset and progression in a transgenic model of familial als. J. Neurosci. Res..

[33] Geloso M.C., Corvino V., Marchese E., Serrano A., Michetti F., D’Ambrosi N. (2017). The dual role of microglia in als: Mechanisms and therapeutic approaches. Front. Aging Neurosci..

[34] Toepfer M., Schroeder M., Klauser A., Lochmüller H., Hirschmann M., Riepl R.L., Pongratz D., Müller-Felber W. (1997). Delayed colonic transit times in amyotrophic lateral sclerosis assessed with radio-opaque markers. Eur. J. Med. Res..

[35] Sejvar J.J., Holman R.C., Bresee J.S., Kochanek K.D., Schonberger L.B. (2005). Amyotrophic lateral sclerosis mortality in the united states, 1979–2001. Neuroepidemiology.

[36] Chiò A., Logroscino G., Hardiman O., Swingler R., Mitchell D., Beghi E., Traynor B.G., Consortium E. (2009). Prognostic factors in als: A critical review. Amyotroph. Lateral Scler..

[37] Del Aguila M.A., Longstreth W.T., McGuire V., Koepsell T.D., van Belle G. (2003). Prognosis in amyotrophic lateral sclerosis: A population-based study. Neurology.

[38] Li H.-F., Wu Z.-Y. (2016). Genotype-phenotype correlations of amyotrophic lateral sclerosis. Transl. Neurodegener..

[39] Brown R.H., Al-Chalabi A. (2017). Amyotrophic lateral sclerosis. N. Engl. J. Med..

[40] Shultz R.B., Zhong Y. (2017). Minocycline targets multiple secondary injury mechanisms in traumatic spinal cord injury. Neural Regen. Res..

[41] Konermann S., Brigham M.D., Trevino A.E., Joung J., Abudayyeh O.O., Barcena C., Hsu P.D., Habib N., Gootenberg J.S., Nishimasu H. (2015). Genome-scale transcriptional activation by an engineered crispr-cas9 complex. Nature.

[42] Lu H., Le W.D., Xie Y.Y., Wang X.P. (2016). Current therapy of drugs in amyotrophic lateral sclerosis. Curr. Neuropharmacol..

[43] Jaiswal M.K. (2019). Riluzole and edaravone: A tale of two amyotrophic lateral sclerosis drugs. Med. Res. Rev..

[44] Bensimon G., Lacomblez L., Meininger V. (1994). A controlled trial of riluzole in amyotrophic lateral sclerosis. Als/riluzole study group. N. Engl. J. Med..

[45] Taylor J.P., Brown R.H., Cleveland D.W. (2016). Decoding als: From genes to mechanism. Nature.

[46] Feigin V.L., Abajobir A.A., Abate K. (2017). Global, regional, and national burden of neurological disorders during 1990–2015, A systematic analysis for the global burden of disease study 2015. Lancet Neurol..

[47] Melzer N., Meuth S.G., Torres-Salazar D., Bittner S., Zozulya A.L., Weidenfeller C., Kotsiari A., Stangel M., Fahlke C., Wiendl H. (2008). A beta-lactam antibiotic dampens excitotoxic inflammatory cns damage in a mouse model of multiple sclerosis. PLoS ONE..

[48] Yimer E.M., Hishe H.Z., Tuem K.B. (2019). Repurposing of the β-lactam antibiotic, ceftriaxone for neurological disorders: A review. Front. Neurosci..

[49] Sun J., Zhan Y., Mariosa D., Larsson H., Almqvist C., Ingre C., Zagai U., Pawitan Y., Fang F. (2019). Antibiotics use and risk of amyotrophic lateral sclerosis in sweden. Eur. J. Neurol..

[50] Waisbren B.A., Cashman N., Schell R.F., Johnson R. (1987). Borrelia burgdorferi antibodies and amyotrophic lateral sclerosis. Lancet.

[51] Oksi J., Marjamäki M., Nikoskelainen J., Viljanen M.K. (1999). Borrelia burgdorferi detected by culture and pcr in clinical relapse of disseminated lyme borreliosis. Ann. Med..

[52] Halperin J.J., Kaplan G.P., Brazinsky S., Tsai T.F., Cheng T., Ironside A., Wu P., Delfiner J., Golightly M., Brown R.H. (1990). Immunologic reactivity against borrelia burgdorferi in patients with motor neuron disease. Arch. Neurol..

[53] Lujia Y., Xin L., Shiquan W., Yu C., Shuzhuo Z., Hong Z. (2014). Ceftriaxone pretreatment protects rats against cerebral ischemic injury by attenuating microglial activation-induced il-1β expression. Int. J. Neurosci..

[54] Abele D.C., Anders K.H. (1990). The many faces and phases of borreliosis. I. Lyme disease. J. Am. Acad. Dermatol..

[55] Lo R., Menzies D.J., Archer H., Cohen T.J. (2003). Complete heart block due to lyme carditis. J. Invasive Cardiol..

[56] Rothstein J.D., Patel S., Regan M.R., Huang Y.H., Bergles D.E., Jin L., Dykes Hoberg M., Vidensky S., Chung D.S. (2005). Beta-lactam antibiotics offer neuroprotection by increasing glutamate transporter expression. Nature.

[57] Thöne-Reineke C., Neumann C., Namsolleck P., Schmerbach K., Krikov M., Schefe J.H., Lucht K., Hörtnagl H., Godes M., Müller S. (2008). The beta-lactam antibiotic, ceftriaxone, dramatically improves survival, increases glutamate uptake and inducesGrinth neurotrophins in stroke. J. Hypertens..

[58] Zumkehr J., Rodriguez-Ortiz C.J., Cheng D., Kieu Z., Wai T., Hawkins C., Kilian J., Lim S.L., Medeiros R., Kitazawa M. (2015). Ceftriaxone ameliorates tau pathology and cognitive decline via restoration of glial glutamate transporter in a mouse model of alzheimer’s disease. Neurobiol. Aging..

[59] Alkasir R., Li J., Li X., Jin M., Zhu B. (2016). Human gut microbiota: The links with dementia development. Protein Cell.

[60] Martella A., Firth M., Taylor B.J.M., Göppert A., Cuomo E.M., Roth R.G., Dickson A.J., Fisher D.I. (2019). Systematic evaluation of crispra and crispri modalities enables development of a multiplexed, orthogonal gene activation and repression system. ACS Synth. Biol..

[61] Cudkowicz M.E., Titus S., Kearney M., Yu H., Sherman A., Schoenfeld D., Hayden D., Shui A., Brooks B., Conwit R. (2014). Safety and efficacy of ceftriaxone for amyotrophic lateral sclerosis: A multi-stage, randomised, double-blind, placebo-controlled trial. Lancet Neurol..

[62] Aliev G., Obrenovich M.E., Reddy V.P., Shenk J.C., Moreira P.I., Nunomura A., Zhu X., Smith M.A., Perry G. (2008). Antioxidant therapy in alzheimer’s disease: Theory and practice. Mini Rev. Med. Chem..

[63] Battaglia G., Riozzi B., Bucci D., Di Menna L., Molinaro G., Pallottino S., Nicoletti F., Bruno V. (2015). Activation of mglu3 metabotropic glutamate receptors enhances gdnf and glt-1 formation in the spinal cord and rescues motor neurons in the sod-1 mouse model of amyotrophic lateral sclerosis. Neurobiol. Dis..

[64] Reaume A.G., Elliott J.L., Hoffman E.K., Kowall N.W., Ferrante R.J., Siwek D.F., Wilcox H.M., Flood D.G., Beal M.F., Brown R.H. (1996). Motor neurons in cu/zn superoxide dismutase-deficient mice develop normally but exhibit enhanced cell death after axonal injury. Nat. Genet..

[65] Scofield M.D., Kalivas P.W. (2014). Astrocytic dysfunction and addiction: Consequences of impaired glutamate homeostasis. Neuroscientist.

[66] Teichberg V.I., Cohen-Kashi-Malina K., Cooper I., Zlotnik A. (2009). Homeostasis of glutamate in brain fluids: An accelerated brain-to-blood efflux of excess glutamate is produced by blood glutamate scavenging and offers protection from neuropathologies. Neuroscientist.

[67] Tikka T., Usenius T., Tenhunen M., Keinänen R., Koistinaho J. (2001). Tetracycline derivatives and ceftriaxone, a cephalosporin antibiotic, protect neurons against apoptosis induced by ionizing radiation. J. Neurochem..

[68] Yacila G., Sari Y. (2014). Potential therapeutic drugs and methods for the treatment of amyotrophic lateral sclerosis. Curr. Med. Chem..

[69] Blacher E., Bashiardes S., Shapiro H., Rothschild D., Mor U., Dori-Bachash M., Kleimeyer C., Moresi C., Harnik Y., Zur M. (2019). Potential roles of gut microbiome and metabolites in modulating als in mice. Nature.

[70] Obrenovich M.E., Palacios H.H., Gasimov E., Leszek J., Aliev G. (2009). The grk2 overexpression is a primary hallmark of mitochondrial lesions during early alzheimer disease. Cardiovasc. Psychiatry Neurol..

[71] Gray M.W. (2017). Lynn margulis and the endosymbiont hypothesis: 50 years later. Mol. Biol. Cell..

[72] Joshi A.U., Saw N.L., Shamloo M., Mochly-Rosen D. (2018). Drp1/fis1 interaction mediates mitochondrial dysfunction, bioenergetic failure and cognitive decline in alzheimer’s disease. Oncotarget.

[73] Brown K.J., Jewells V., Herfarth H., Castillo M. (2010). White matter lesions suggestive of amyotrophic lateral sclerosis attributed to celiac disease. Am. J. Neuroradiol..

[74] Tanaka K., Kanno T., Yanagisawa Y., Yasutake K., Inoue S., Hirayama N., Ikeda J.E. (2014). A novel acylaminoimidazole derivative, wn1316, alleviates disease progression via suppression of glial inflammation in als mouse model. PLoS ONE.

[75] Nau R., Prange H.W., Muth P., Mahr G., Menck S., Kolenda H., Sörgel F. (1993). Passage of cefotaxime and ceftriaxone into cerebrospinal fluid of patients with uninflamed meninges. Antimicrob. Agents Chemother..

[76] Aliev G., Liu J., Shenk J.C., Fischbach K., Pacheco G.J., Chen S.G., Obrenovich M.E., Ward W.F., Richardson A.G., Smith M.A. (2009). Neuronal mitochondrial amelioration by feeding acetyl-l-carnitine and lipoic acid to aged rats. J. Cell. Mol. Med..

[77] Mark L.P., Prost R.W., Ulmer J.L., Smith M.M., Daniels D.L., Strottmann J.M., Brown W.D., Hacein-Bey L. (2001). Pictorial Review of Glutamate Excitotoxicity: Fundamental Concepts for Neuroimaging. AJNR Am. J. Neuroradiol..

[78] Tremlett H., Bauer K.C., Appel-Cresswell S., Finlay B.B., Waubant E. (2017). The gut microbiome in human neurological disease: A review. Ann. Neurol..

[79] Gurney M.E., Pu H., Chiu A.Y., Dal Canto M.C., Polchow C.Y., Alexander D.D., Caliendo J., Hentati A., Kwon Y.W., Deng H.X. (1994). Motor neuron degeneration in mice that express a human Cu,Zn superoxide dismutase mutation. Science.

[80] Thany S.H. (2010). Neonicotinoid insecticides: Historical evolution and resistance mechanisms. Adv. Exp. Med. Biol..

[81] Simon-Delso N., Amaral-Rogers V., Belzunces L.P., Bonmatin J.M., Chagnon M., Downs C., Furlan L., Gibbons D.W., Giorio C., Girolami V. (2015). Systemic insecticides (neonicotinoids and fipronil): Trends, uses, mode of action and metabolites. Environ. Sci. Pollut. Res Int..

[82] Sundh U.M., Andersoon C., Rosén J., Fonnum F., Knudsen I., Sippola S. (2007). Analysis, Occurrence and Toxicity of BMAA.

[83] Seifert J., Stollberg J. (2005). Antagonism of a neonicotinoid insecticide imidacloprid at neuromuscular receptors. Env. Toxicol. Pharmacol..

[84] Li H., Haixing G., Dandan C., Yusheng X. (2008). A high γ-aminobutyric acid-producing lactobacillus *brevis* isolated from chinese traditional paocai. Ann. Microbiol..

[85] Vlachojannis C., Zimmermann B.F., Chrubasik-Hausmann S. (2015). Quantification of anthocyanins in elderberry and chokeberry dietary supplements. Phytother. Res..

[86] Patel P., Julien J.P., Kriz J. (2015). Early-stage treatment with withaferin a reduces levels of misfolded superoxide dismutase 1 and extends lifespan in a mouse model of amyotrophic lateral sclerosis. Neurotherapeutics.

[87] Iketani M., Ohsawa I. (2017). Molecular hydrogen as a neuroprotective agent. Curr. Neuropharmacol..

[88] Zhang Y., Li H., Yang C., Fan D.F., Guo D.Z., Hu H.J., Meng X.E., Pan S.Y. (2016). Treatment with hydrogen-rich saline delays disease progression in a mouse model of amyotrophic lateral sclerosis. Neurochem. Res..

[89] Şahin S., Gürgen S.G., Yazar U., İnce İ., Kamaşak T., Acar Arslan E., Diler Durgut B., Dilber B., Cansu A. (2019). Vitamin d protects against hippocampal apoptosis related with seizures induced by kainic acid and pentylenetetrazol in rats. Epilepsy Res..

[90] Pascuzzi R.M., Shefner J., Chappell A.S., Bjerke J.S., Tamura R., Chaudhry V., Clawson L., Haas L., Rothstein J.D. (2010). A phase ii trial of talampanel in subjects with amyotrophic lateral sclerosis. Amyotroph. Lateral Scler..

[91] Sanchez S., Demain A.L. (2008). Metabolic regulation and overproduction of primary metabolites. Microb. Biotechnol..

[92] Libonati L., Onesti E., Gori M.C., Ceccanti M., Cambieri C., Fabbri A., Frasca V., Inghilleri M. (2017). Vitamin d in amyotrophic lateral sclerosis. Funct. Neurol..

[93] Bikard D., Euler C.W., Jiang W., Nussenzweig P.M., Goldberg G.W., Duportet X., Fischetti V.A., Marraffini L.A. (2014). Exploiting crispr-cas nucleases to produce sequence-specific antimicrobials. Nat. Biotechnol..

[94] Gianforcaro A., Solomon J.A., Hamadeh M.J. (2019). Vitamin d(3) at 50x ai attenuates the decline in paw grip endurance, but not disease outcomes, in the g93a mouse model of als, and is toxic in females. PLoS ONE.

[95] Papapetropoulos S. (2007). Is there a role for naturally occurring cyanobacterial toxins in neurodegeneration? The beta-n-methylamino-l-alanine (bmaa) paradigm. Neurochem. Int..

[96] Vyas K., Weiss J.H. (2009). Bmaa—An unusual cyanobacterial neurotoxin. Amyotroph. Lateral Scler..

[97] Marietta E., Horwath I., Taneja V. (2018). Microbiome, immunomodulation, and the neuronal system. Neurotherapeutics.

[98] Wright M.L., Fournier C., Houser M.C., Tansey M., Glass J., Hertzberg V.S. (2018). Potential role of the gut microbiome in als: A systematic review. Biol. Res. Nurs..

[99] Chiu A.S., Gehringer M.M., Braidy N., Guillemin G.J., Welch J.H., Neilan B.A. (2012). Excitotoxic potential of the cyanotoxin β-methyl-amino-l-alanine (bmaa) in primary human neurons. Toxicon.

[100] Aliev G., Gasimov E., Obrenovich M.E., Fischbach K., Shenk J.C., Smith M.A., Perry G. (2008). Atherosclerotic lesions and mitochondria dna deletions in brain microvessels: Implication in the pathogenesis of alzheimer’s disease. Vasc. Health Risk Manag..

[101] Goutman S.A., Brown M.B., Cudkowicz M., Atassi N., Feldman E.L. (2019). Als/surv: A modification of the cafs statistic. Amyotroph. Lateral Scler. Front. Degener..

[102] Guo H., Lai L., Butchbach M.E., Stockinger M.P., Shan X., Bishop G.A., Lin C.L. (2003). Increased expression of the glial glutamate transporter eaat2 modulates excitotoxicity and delays the onset but not the outcome of als in mice. Hum. Mol. Genet..

[103] Rothstein J.D., Van Kammen M., Levey A.I., Martin L.J., Kuncl R.W. (1995). Selective loss of glial glutamate transporter glt-1 in amyotrophic lateral sclerosis. Ann. Neurol..

[104] Brouwers N., Van Cauwenberghe C., Engelborghs S., Lambert J.C., Bettens K., Le Bastard N., Pasquier F., Montoya A.G., Peeters K., Mattheijssens M. (2012). Alzheimer risk associated with a copy number variation in the complement receptor 1 increasing c3b/c4b binding sites. Mol. Psychiatry..

[105] Kurosawa K., Misu T., Takai Y., Sato D.K., Takahashi T., Abe Y., Iwanari H., Ogawa R., Nakashima I., Fujihara K. (2015). Severely exacerbated neuromyelitis optica rat model with extensive astrocytopathy by high affinity anti-aquaporin-4 monoclonal antibody. Acta Neuropathol. Commun..

[106] Trotti D., Rolfs A., Danbolt N.C., Brown R.H., Hediger M.A. (1999). Sod1 mutants linked to amyotrophic lateral sclerosis selectively inactivate a glial glutamate transporter. Nat. Neurosci..

[107] Kumar D.K., Choi S.H., Washicosky K.J., Eimer W.A., Tucker S., Ghofrani J., Lefkowitz A., McColl G., Goldstein L.E., Tanzi R.E. (2016). Amyloid-β peptide protects against microbial infection in mouse and worm models of alzheimer disease. Sci. Transl. Med..

[108] Welling M., Nabuurs R.J., van der Weerd L. (2015). Potential role of antimicrobial peptides in the early onset of alzheimer’s disease. Alzheimer’s Dement..

[109] Obrenovich M., Tabrez S., Siddiqui B., McCloskey B., Perry G. (2020). The microbiota-gut-brain axis-heart shunt part ii: Prosaic foods and the brain-heart connection in alzheimer disease. Microorganisms.

[110] Yamamoto Y. (2002). Pcr in diagnosis of infection: Detection of bacteria in cerebrospinal fluids. Clin. Diagn. Lab. Immunol..

[111] Spiller K.J., Restrepo C.R., Khan T., Dominique M.A., Fang T.C., Canter R.G., Roberts C.J., Miller K.R., Ransohoff R.M., Trojanowski J.Q. (2018). Microglia-mediated recovery from als-relevant motor neuron degeneration in a mouse model of tdp-43 proteinopathy. Nat. Neurosci..

[112] Volonté C., Amadio S., Fabbrizio P., Apolloni S. (2019). Functional microglia neurotransmitters in amyotrophic lateral sclerosis. Semin. Cell Dev. Biol..

[113] Evans M.C., Couch Y., Sibson N., Turner M.R. (2013). Inflammation and neurovascular changes in amyotrophic lateral sclerosis. Mol. Cell. Neurosci..

[114] Zhang D., Hu X., Qian L., O’Callaghan J.P., Hong J.S. (2010). Astrogliosis in cns pathologies: Is there a role for microglia?. Mol. Neurobiol..

[115] Volterra A., Meldolesi J. (2005). Astrocytes, from brain glue to communication elements: The revolution continues. Nat. Rev. Neurosci..

[116] Manabe Y., Nagano I., Gazi M.S., Murakami T., Shiote M., Shoji M., Kitagawa H., Setoguchi Y., Abe K. (2002). Adenovirus-mediated gene transfer of glial cell line-derived neurotrophic factor prevents motor neuron loss of transgenic model mice for amyotrophic lateral sclerosis. Apoptosis.

[117] Bender K.O., Garland M., Bogyo M. (2016). Response to comment on “A small-molecule antivirulence agent for treating clostridium difficile infection”. Sci. Transl. Med..

[118] Figueira I., Menezes R., Macedo D., Costa I., Santos C. (2017). Claudiapolyphenols beyond barriers: A glimpse *Uddin* into the brain current neuropharmacology. Curr. Neuropharmacol..

[119] Korkmaz O.T., Aytan N., Carreras I., Choi J.K., Kowall N.W., Jenkins B.G., Dedeoglu A. (2014). 7,8-dihydroxyflavone improves motor performance and enhances lower motor neuronal survival in a mouse model of amyotrophic lateral sclerosis. Neurosci. Lett..

[120] Mendonça-Filho R.R., Rodrigues I.A., Alviano D.S., Santos A.L., Soares R.M., Alviano C.S., Lopes A.H., Maria do Socorro S.R. (2004). Leishmanicidal activity of polyphenolic-rich extract from husk fiber of Cocos nucifera Linn. (Palmae). Res. Microbiol..

[121] Hosaka T., Yamashita T., Tamaoka A., Kwak S. (2019). Extracellular rnas as biomarkers of sporadic amyotrophic lateral sclerosis and other neurodegenerative diseases. Int. J. Mol. Sci..

[122] Petimar J., O’Reilly É., Adami H.O., van den Brandt P.A., Buring J., English D.R., Freedman D.M., Giles G.G., Håkansson N., Kurth T. (2019). Coffee, tea, and caffeine intake and amyotrophic lateral sclerosis mortality in a pooled analysis of eight prospective cohort studies. Eur. J. Neurol..

[123] Liu T., Wang T., Li X., Liu X. (2008). Improved heterologous gene expression in trichoderma reesei by cellobiohydrolase i gene (cbh1) promoter optimization. Acta Biochim. Biophys Sin..

[124] Yadav R., Kumar V., Baweja M., Shukla P. (2018). Gene editing and genetic engineering approaches for advanced probiotics: A review. Crit. Rev. Food Sci. Nutr..

[125] Lubitz D., Wendisch V.F. (2016). Ciprofloxacin triggered glutamate production by corynebacterium glutamicum. BMC Microbiol..

